# Characterization of killer immunoglobulin-like receptor genetics and comprehensive genotyping by pyrosequencing in rhesus macaques

**DOI:** 10.1186/1471-2164-12-295

**Published:** 2011-06-07

**Authors:** Anna J Moreland, Lisbeth A Guethlein, R Keith Reeves, Karl W Broman, R Paul Johnson, Peter Parham, David H O'Connor, Benjamin N Bimber

**Affiliations:** 1 Department of Pathology and Laboratory Medicine, University of Wisconsin-Madison, Madison, Wisconsin 53706, USA; 2 Departments of Structural Biology and Microbiology & Immunology, Stanford University School of Medicine, Stanford, CA 94305, USA; 3 Division of Immunology, New England Primate Research Center, Harvard Medical School, Southborough, MA 01772, USA; 4 Department of Biostatistics and Medical Informatics, University of Wisconsin-Madison, Madison, Wisconsin 53706, USA; 5 Wisconsin National Primate Research Center, University of Wisconsin-Madison, Madison, Wisconsin 53706, USA

## Abstract

**Background:**

Human killer immunoglobulin-like receptors (KIRs) play a critical role in governing the immune response to neoplastic and infectious disease. Rhesus macaques serve as important animal models for many human diseases in which KIRs are implicated; however, the study of KIR activity in this model is hindered by incomplete characterization of *KIR *genetics.

**Results:**

Here we present a characterization of *KIR *genetics in rhesus macaques (*Macaca mulatta)*. We conducted a survey of *KIRs *in this species, identifying 47 novel full-length *KIR *sequences. Using this expanded sequence library to build upon previous work, we present evidence supporting the existence of 22 *Mamu-KIR *genes, providing a framework within which to describe macaque *KIRs*. We also developed a novel pyrosequencing-based technique for *KIR *genotyping. This method provides both comprehensive *KIR *genotype and frequency estimates of transcript level, with implications for the study of *KIRs *in all species.

**Conclusions:**

The results of this study significantly improve our understanding of macaque *KIR *genetic organization and diversity, with implications for the study of many human diseases that use macaques as a model. The ability to obtain comprehensive KIR genotypes is of basic importance for the study of KIRs, and can easily be adapted to other species. Together these findings both advance the field of macaque KIRs and facilitate future research into the role of KIRs in human disease.

## Background

Killer immunoglobulin-like receptors (KIRs) are a highly polymorphic family of cell surface receptors expressed on natural killer (NK) cells and a subset of T-lymphocytes [1-3]. KIR mediated signaling plays a key role in the identification of foreign cells and the antiviral response [4-9]. The best characterized KIR ligands are major histocompatibility complex class I (MHC-I) molecules, although ligands have not been identified for all KIRs [10,11]. Because both KIRs and MHC-I are highly polymorphic, host genotype plays an important role in KIR function.

*KIR *genetic diversity can be described in terms of polymorphism and polygenicity. To date, there are 15 *KIR *genes described in humans [12]. The number of *KIR *genes varies between individuals, with 7-12 genes per haplotype [13,14]. Because the protein product of each *KIR *gene generally binds a unique set of ligands, the subset of KIRs encoded by an individual dictates the potential KIR interactions that can occur. In addition to variation in gene content between haplotypes, there is allelic polymorphism within each *KIR *gene [13]. Broadly speaking, the allotypic variants encoded by a *KIR *gene bind the same subset of MHC-I ligands, although exceptions do exist [15]. Distinct KIR allotypes can have differing binding affinities for particular MHC-I allotypes. These differences in KIR/MHC-I binding affinity can alter KIR signaling and NK cell activity [16]. In addition to *KIR *genotype, *MHC-I *genotype must be considered since it determines the set of available KIR ligands and since it is possible to express a KIR with or without its cognate MHC-I molecule [10,17].

Specific *KIR/MHC-I *genotypes have been implicated as a factor contributing to the immune control of multiple human diseases including hepatitis C virus, human papilloma virus, malaria, and human immunodeficiency virus (HIV) [6-9,18]. One of the best-studied examples of *KIR/MHC-I *genetics and disease is that of *KIR3DL1/KIR3DS1 *and *HLA-Bw4 *in HIV infection. Individuals who express specific *KIR3DL1/KIR3DS1 *alleles in combination with certain *HLA-B *alleles containing the Bw4 motif show slower progression to AIDS [8,9]. This genetic association has more recently been supported by functional data demonstrating that NK cells expressing KIR3DS1 have increased anti-HIV activity against target cells expressing HLA-Bw4, although the underlying mechanism remains to be elucidated [19].

Despite advances in our understanding of KIR biology, the mechanisms through which specific KIR/MHC-I combinations influence disease progression are not fully understood. This is at least partially due to the complexity of *KIR/MHC-I *genotypes and difficulty in identifying *KIR/MHC-I *matched cohorts. Rhesus macaques (*Macaca mulatta) *are an established and widely used experimental model system for many human diseases, including immunodeficiency virus [20]. The advantages of studying infectious disease in rhesus macaques include the ability to manipulate the dose, route, and strain of the infectious agent, as well as the ability to analyze specimens from defined time points. For the study of KIR activity, perhaps the most important advantage is the ability to select subjects based on genetics. This benefit is evidenced by the work in macaques to elucidate the role of the cytotoxic T-lymphocyte (CTL) response in immunodeficiency viral infection, which is also heavily dependent on host genetics [21,22].

Macaque KIRs have received less study than human KIRs. While previous work shows that macaque KIRs have structure and genomic organization similar to human KIRs, and suggests that they play a similar functional role, these studies also demonstrate that there has been considerable evolution within macaque KIRs since the species diverged [23]. While 15 genes have been described in humans, the number and identity of the KIR genes present in macaques is distinct. Developing an understanding of the KIR genes present in this species and an overall assessment of KIR genetic diversity is a matter of practical importance for the use of macaques as a model for KIR function. Using cDNA sequences, an initial model for macaque *KIR *genetic organization was formed containing 18 putative KIR groups [23,24]. In addition to the sequence of *KIR *transcripts, the genomic sequence of one rhesus macaque *KIR *haplotype has been described [25]. More recent studies have added to the total number of described macaque *KIR *sequences [23,24,26-28]. With more sequence data available, phylogenetic relationships became clearer, and the model of macaque KIR genetics has been refined. This body of work has been used to create a model for macaque KIR genetic organization and to develop a formal system of nomenclature (Guethlein et al, in preparation).

Here we present the results of a survey of rhesus macaque *KIR *genetics. Using full-length cloning, we identified 47 novel full-length rhesus macaque KIRs, substantially increasing the library of known sequences. Using this expanded library, we performed phylogenetic analysis supporting the existence of 22 rhesus macaque *KIR *genes. Together with previously published KIR sequences, this provides a framework with which to describe *KIR *genetics in this species. In addition to improving our understanding of macaque *KIRs *at the population level, we developed a novel pyrosequencing-based approach for *KIR *genotyping. This technique provides both comprehensive *KIR *genotyping and frequency estimates for expression of each *KIR *transcript. The findings presented here, along with the novel techniques set forth, should serve as a foundation for further research on rhesus *KIR *genetics and for defining KIR function in this important animal model.

## Methods

### Animal Care and Specimen Processing

Animals used in this study were housed and cared for by the trained veterinary staff at the Wisconsin National Primate Research Center (WNPRC) or the New England Primate Research Center (NEPRC). All procedures were approved by the host institution's Animal Care and Use Committee. Nucleic acid was obtained from peripheral blood mononuclear cells (PBMC) or purified natural killer (NK) cells, as indicated. RNA purification was accomplished using either the MagnaPure LC Total Nucleic Acid Purification kit (Roche, Branford, CT) or the DNA/RNA Allprep Kit (QIAGEN, Valencia, CA) according to the manufacturer's instructions.

NK cells were isolated from whole PBMC by negative magnetic bead fractionation. First, PBMC were incubated for 20 minutes at room temperature in 0.1% BSA/PBS with a cocktail of cross-reactive human monoclonal IgG antibodies composed of the following: anti-CD3 (clone SP34-2, BD Biosciences, La Jolla, CA), anti-CD14 (clone M5E2, BD Biosciences), anti-CD40 (clone 5C3, BD Biosciences), and anti-CD66/CEACAM (clone TET2, AbCam, Cambridge, MA or Santa Cruz Biotechnology, Santa Cruz, CA). Next, antibody-coated PBMC were washed and resuspended in 0.1% BSA/PBS then incubated for 35 minutes at room temperature with Pan-IgG Dynabeads (Dynal Biotech, Norway) at a 4:1 bead-to-cell ratio. The suspension was then placed on a Dynal magnet and the unbound cells were collected. Purity was assessed by flow cytometry with >90% of collected cells routinely bearing an NK cell phenotype of NKG2A^+^CD8^+^CD3^-^. All acquisitions were made on a FACSCalibur (BD Biosciences) and analyzed using FlowJo software (Tree Star Inc., Ashland, OR).

### Full-Length cDNA Cloning and Sequencing

First-strand cDNA was synthesized using the Superscript III First-Strand One-Step RT-PCR kit (Invitrogen, Carlsbad, CA) according to the manufacturer's instructions. PCR amplification was performed using Phusion high-fidelity polymerase (New England Biolabs, Ipswich, MA) and the following external primers: 5'-CAGCACCATGTCGCTCAT-3' and 5'-GGGGTCAAGTGAAGTGGAGA-3'. PCR conditions were: 98°C for 30 s, 28 cycles of 98°C for 5 s, 63°C for 1 s, 72°C for 20 s, and a final extension at 72°C for 5 min. PCR products were cloned into pCR-Blunt TOPO (Invitrogen, Carlsbad, CA) and bidirectionally sequenced using DYEnamic ET Terminator cycle sequencing kit (GE Healthcare, Piscataway, NJ). Internal primers used in sequencing were 5'-AACCTTCCCTCCTGGCC-3 and 5'-TTGGTTCAGTGGGTGAAGGCCAA-3.' CodonCode Aligner (CodonCode Corporation, Dedham, MA) was used for sequence analysis, and in order to minimize error introduced by PCR artifacts, novel alleles were only included when three or more identical full-length cDNA clones were observed. Novel sequences have been deposited in Genbank (Additional file 1, Table S1). Novel full-length sequences were assigned formal names through the Immuno Polymorphism Database [29].

### Phylogenetic Analysis

The cDNA sequences obtained in this analysis and from previous studies [23,25,27,28,30] were aligned using Clustal X [31] and manually corrected in BioEdit (Ibis Therapeutics, Carlsbad, CA). Phylogenetic trees for the complete dataset were made using both neighbor-joining (1000 replicates, pairwise deletion, Tamura-Nei, in MEGA4) and parsimony (1000 replicates in Paup). These trees were used to divide the sequences into *Mamu-KIR3DL20, -KIR2DL04, -KIR1D*, and *-KIR3D *(lineage II) groups. Subsequent analysis was restricted to the lineage II dataset using the same methods. Alleles of *Mamu-KIR3DL11, -KIR3DS01, -KIR3DS03, -KIR3DSW07*, and -*KIR3DSW08 *were not obtained in this study and sequences for these genes were taken from GenBank [23,27,30,32].

A dataset containing consensus sequences for each of the lineage II KIR genes was constructed using all known sequences to compute the consensus sequence. For groups containing only two allotypes, one of the allotypes was chosen to represent the consensus. The sequences used are indicated in Additional file 1, Figure S1. The individual extracellular Ig domains (D0, D1, and D2) were analyzed separately using the methods described above. The stem, transmembrane and cytoplasmic tails were not included in this analysis as previous reports have shown that the stem, transmembrane, and tail of macaque activating KIRs share similarity to KIR2DL4 and are distinct [23].

### PCR Amplification and Pyrosequencing

As with our full-length products, cDNA was synthesized using the Superscript III First-Strand One-Step RT-PCR kit (Invitrogen, Carlsbad, CA). cDNA-PCR amplicons spanning 623 base pairs of the D1 and D2 domains were synthesized using Phusion high-fidelity polymerase (New England Biolabs, Ipswich, MA). Each PCR primer contained a target-specific sequence, an MID tag, and an adapter sequence (Additional file 1, Table S2). PCR conditions were: 98°C for 30 s, 28-33 cycles of 98°C for 5 s, 61°C for 1 s, 72°C for 20 s, and a final extension at 72°C for 5 min. cDNA-PCR product purification was accomplished using Ampure XP beads (Agencourt, Beverly, MA) according to the manufacturer's instructions. Amplicons were then normalized to equimolar concentrations and grouped into pools of twelve samples for Titanium amplicon pyrosequencing. Emulsion PCR, Roche/454 Titanium amplicon pyrosequencing, image processing, and base calling were performed according to the manufacturer's instructions (Roche/454 Life Sciences, Branford, CT) at the University of Illinois at Urbana-Champaign High-Throughput Sequencing Center. Each pool of twelve samples was sequenced in one-sixteenth of a 70 × 75 PicoTiterPlate.

### Sequence Analysis

Pyrosequencing flowgram data was processed using a custom analysis pipeline. Briefly, data were trimmed by sequence quality and aligned against a reference database of all known macaque *KIR *sequences using the Mosaik aligner (http://code.google.com/p/mosaik-aligner/). The reference library of *KIR *sequences was obtained from the Immuno Polymorphism Database [29]. Polymorphisms between reads and the reference sequences were scored with custom scripts that utilized Samtools and BioPerl [33,34]. The source code for this pipeline can be obtained from a subversion repository (https://hedgehog.fhcrc.org/tor/stedi/trunk/server/customModules/SequenceAnalysis). The pipeline itself has been integrated into the LabKey Software platform as the SequenceAnalysis module, which provides a graphical, web-based platform to initiate analysis pipelines and view results. LabKey is a free, open source software package available at http://www.labkey.org. Sanger sequence data was analyzed using CodonCode Aligner (CodonCode Corporation, Dedham, MA).

In order to reduce errors introduced by PCR artifacts, *KIR *sequences were only considered to be present in an animal if they represented one percent or more of total *KIR *sequence reads from that animal. In order to identify novel alleles not present in the reference library, unaligned sequences were then assembled in CodonCode Aligner at 100% identity. BLAST analysis was performed for the resulting contigs against a database of published *Mamu-KIR *sequences. Unaligned sequences were deemed novel when they represented at least one percent of total sequence reads from at least one animal and did not represent a potential insertion/deletion error in the pyrosequencing base-calling.

## Results

### Rhesus macaque KIR diversity

*KIR *genotype has a significant influence on NK cell activity. Studying the role of macaque *KIR *genetics in disease pathogenesis first requires an understanding of total diversity within the population and a framework within which to describe it. To date, 149 distinct full-length *Mamu-KIR *sequences have been deposited in Genbank. By comparison, there are more than 615 distinct *KIR *alleles identified in humans--more than four times as many alleles as have been identified to date in rhesus macaques [35]. Thus, the previously catalogued alleles likely represent only a fraction of total *Mamu-KIR *diversity. In order to expand the repertoire of known *Mamu-KIR *alleles, we performed full-length cloning and sequencing of *KIR *alleles from 17 Indian rhesus macaques. These animals yielded 47 novel *KIR *alleles, a substantial expansion of documented rhesus macaque *KIR *diversity (Additional file 1, Table S1). The novel sequences have been assigned names following the KIR genes and nomenclature conventions established in the Non-human Primate KIR Nomenclature Report (Guethlein et al, in preparation)^1^.

**Figure 1:**
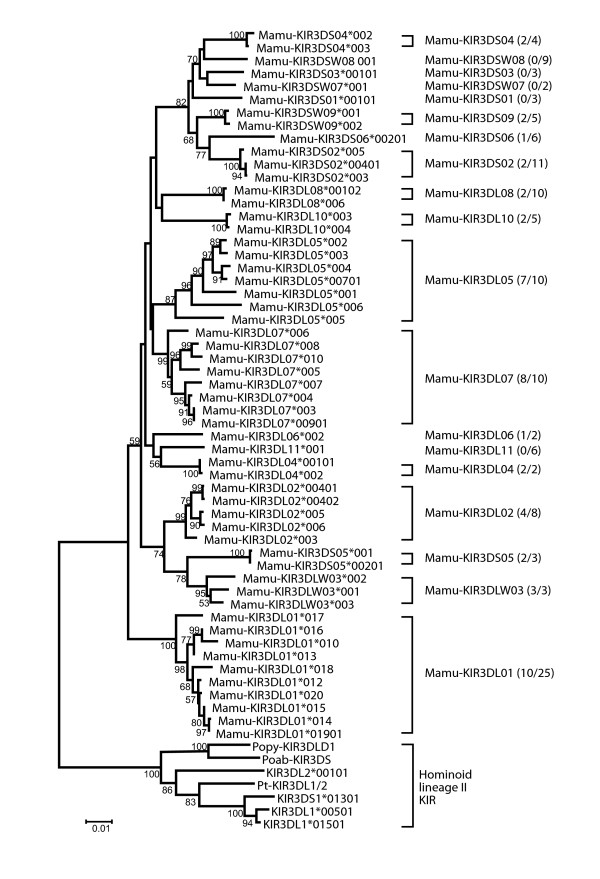
**Rhesus macaque lineage II *KIR *alleles form 19 distinct groups**. Neighbor-joining and parsimony analysis was performed using predicted amino-acid sequences for the dataset comprising the lineage II sequences obtained in this study. The neighbor-joining tree is shown, which was not significantly different from the parsimony tree [data not shown]. Bootstrap support of greater than 50% is indicated. Representative hominoid lineage II *KIR *were used as an outgroup. For each gene, the numbers in parentheses indicate the total number of alleles discovered in this study compared with the total number known.

As a first step in the analysis of the rhesus macaque sequences recovered from cloning, phylogenetic trees based on the full-length sequences were constructed. Human *KIRs *have been divided by phylogenetic analyses into four lineages^2^. Macaque KIRs assorted into groups matching these lineages. The sequences assorted into *Mamu-KIR2DL04 *(lineage I), *-KIR3D *(lineage II), *-KIR1D *(lineage III), and *-KIR3DL20 *(lineage V), with the majority of sequences resembling human lineage II, in accordance with previous publications [25,30,36]. Relatively little allelic variation was observed within the *Mamu-KIR2DL04, -KIR3DL20*, and -*KIR1D *groups (data not shown). In contrast, the *Mamu-KIR3D *lineage II sequences were highly diverse. Our sequences assorted into 19 distinct groups corresponding to 19 of the rhesus macaque lineage II genes (Figure 1 and Additional file 1, Figure S1). An important point to note is that rather than increasing the number of detected *KIR *genes, most novel sequences share strong similarity with established macaque *KIR *genes. Therefore, while there are likely a large number of undiscovered macaque *KIR *alleles, the most common macaque *KIR *genes may now have been identified.

**Figure 2:**
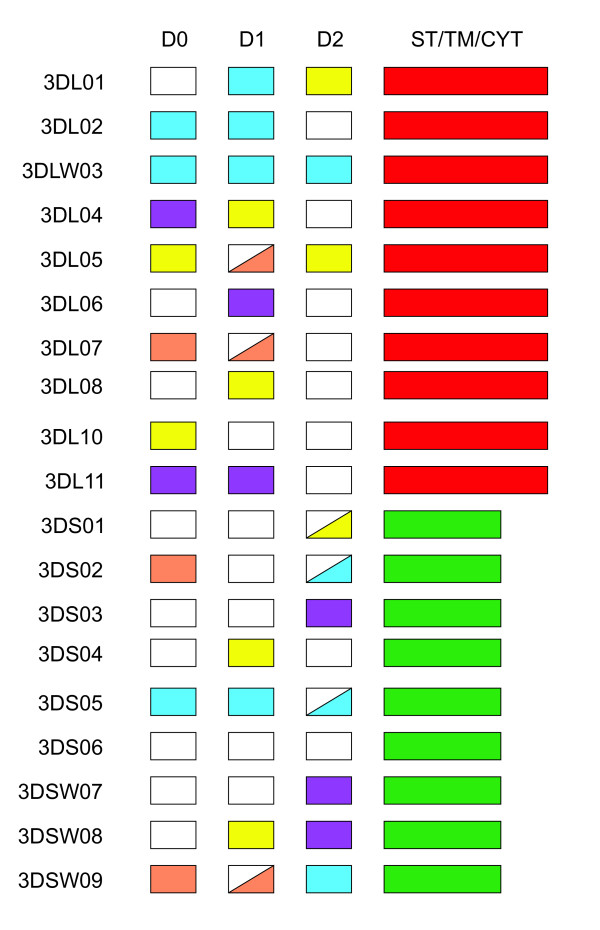
**Domain shuffling has acted to form rhesus macaque *KIR *genes**. The results of a domain-by-domain phylogenetic analysis are shown schematically. Predicted amino-acid consensus sequences for each of the genes were used to form both neighbor-joining and parsimony trees as described in the Methods section. The sequences encoding the three extracellular Ig-like domains were analyzed separately. Boxes are colored for the D0, D1, and D2 domains where the grouping was supported by >50% bootstrap support in the phylogenetic analyses. Boxes that are not completely colored represent cases where support of >50% was only found in the neighbor-joining analysis. Long cytoplasmic tails are colored red and short tails are colored green. The domains colored white were not resolved into any group in the analysis and should not be interpreted as being closely related to each other. The stem, transmembrane and cytoplasmic tails were grouped as either long or short by inspection of the alignment. D0, D1, and D2 denote the Ig-like domains. ST/TM/CYT denotes the stem, transmembrane, and cytoplasmic tail domains.

There is evidence for extensive recombination among the *Mamu-KIR *sequences described here. To identify recombination events affecting individual alleles, sequences were analyzed with the RDP (recombinant detection program) package and inspected manually (data not shown). In addition, domain-by-domain phylogenetic analysis using consensus sequences for each gene revealed extensive domain sharing between macaque *KIR *genes (Figure 2). This suggests that the genes are themselves products of ancient duplication and recombination events. This mechanism for the generation of *KIR *genes is consistent with observations of *KIRs *in other species [37]. Pairs of KIRs with similar extracellular domains but differing cytoplasmic tails have been observed in other species, with human KIR3DL1/3DS1 being a notable example [38]. In this analysis we also found such pairing, with the best-matched pair being *Mamu-KIR3DLW03 *and *Mamu-KIR3DS05*. Other pairs were identified that were similar in two of the three extracellular Ig-like domains, for example *Mamu-KIR3DL07 *and *Mamu-KIR3DSW09*. Cytoplasmic domains were not included in this analysis, although it should be noted that macaque long and short cytoplasmic tails are phylogenetically distinct, with the latter resembling the tail of human KIR2DL4 [23].

### Comprehensive KIR genotyping by pyrosequencing

The expanded library of macaque *KIR *sequences described in this publication and others has enabled the creation of a framework to describe macaque KIRs, and its phylogenetic analysis suggests that the most common *KIR *genes have been identified [24-28,30,32]. For the study of KIRs in disease pathogenesis, one of the most basic requirements is the ability to identify the KIRs expressed by individual subjects. *KIRs *pose many challenges for genotyping: each subject expresses a distinct number of KIRs, there is extensive sequence homology between *KIR *transcripts, splice variants may be functionally significant, and pseudogenes are common. The cloning-based strategy we employed for allele discovery can be used to provide genotypes of individual subjects; however, this method is labor intensive and will frequently miss low-abundance transcripts due to the limited number of clones examined. Techniques such as sequence-specific PCR (SSP) can be used to identify the presence or absence of a particular gene or allele; however, sequence homology between *KIRs *complicates primer design, and a large number of primer pairs would be required for comprehensive genotyping.

**Figure 3:**
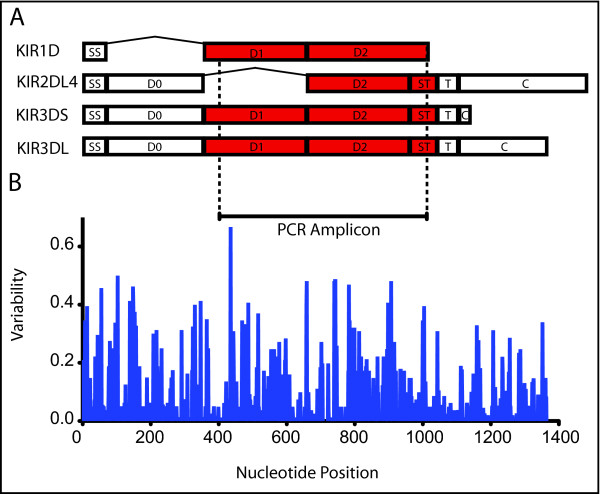
**KIR 454 Titanium Amplicon Primer Design**. A) Schematic representation of macaque KIR3D and KIR2D molecules showing domain structure. B) Variability plot for a cDNA sequence alignment of all published *KIR3DL *alleles. PCR primer sites are indicated along with the region amplified by PCR. Note that 454 sequencing reads span the D1, D2, and stem (ST) regions. The signal sequence (SS), D0, transmembrane region (T), and cytoplasmic region (C) are not amplified by our PCR primers.

**Figure 4:**
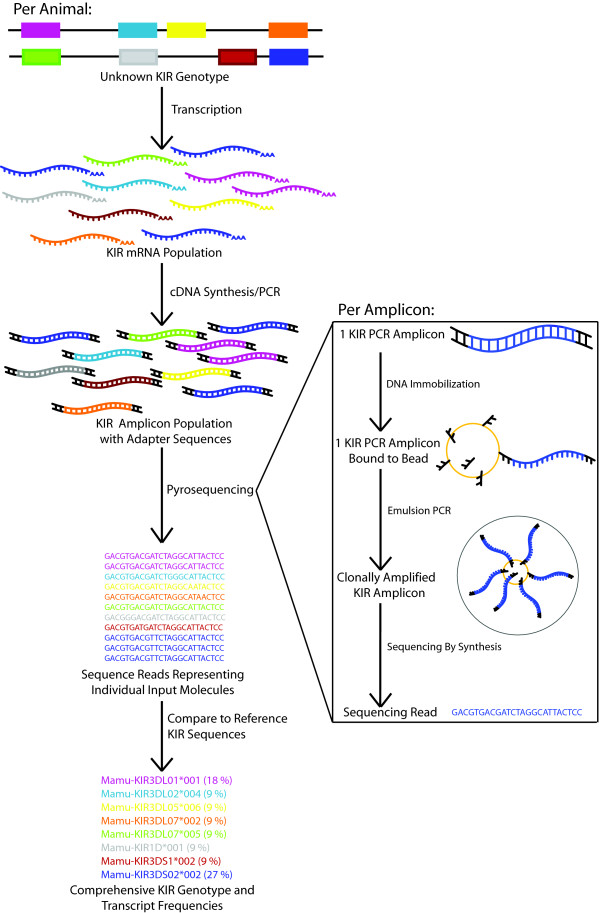
**Outline of KIR genotyping strategy**. The input material is isolated RNA, which will represent all KIRs transcribed by the subject. PCR is performed using conserved KIR-specific primers that add adapter tags. The PCR products are pyrosequenced, producing 1000s of reads, with each read representing a single input molecule. These reads are compared against a reference database of macaque KIR sequences. This analysis produces a list of all KIRs detected per subject, including the relative frequency of each KIR.

To overcome these limitations, we developed a novel sequence-based typing approach. Recent developments in Roche/454 Titanium pyrosequencing technology have enabled sequencing of cDNA-PCR amplicons with high sensitivity and throughput. To adapt this approach for *KIR *genotyping, we first identified primer sites that are highly conserved across all published *Mamu-KIR3D *sequences (Figure 3). These primers amplify a region of 623 bp spanning the majority of D1, all of D2, and part of the stem region. This amplicon was selected to span a polymorphic region of the transcript and to maximize conservation of primers. These primer sites are also conserved amongst most published *Mamu-KIR1D *alleles--although they will not amplify *Mamu-KIR2DL04 *sequences, as these lack the D1 region and therefore the 5' primer-binding site. These primers were used for PCR on cDNA derived from total RNA, producing amplicons representing the distinct *Mamu-KIR3D *and -*KIR1D *transcripts expressed by each subject. Pyrosequencing of these amplicons produces clonal sequence reads corresponding to individual input transcripts. Collectively, these reads represent the KIRs expressed by the subject, with the exception of *Mamu-KIR2DL04*. A schematic of this process is shown in Figure 4.

**Figure 5:**
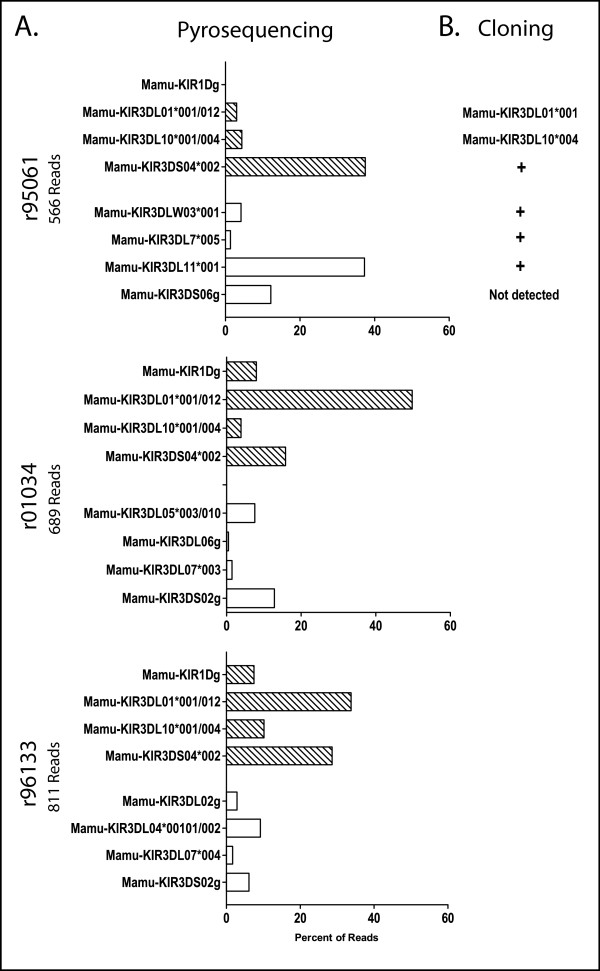
**Differential KIR expression between subjects with a common KIR haplotype**. A) *KIR *genotypes obtained by pyrosequencing are shown for three rhesus macaque half-siblings. The relative expression level of each detected *KIR *allele is shown. Striped bars indicate alleles present on their shared *KIR *haplotype. The number of pyrosequencing reads is shown to the left of each graph. B) For r95061, the *KIR *alleles detected by cDNA cloning are shown. A total of 101 clones were examined. If indicated, the allele detected is listed. A plus sign indicates the genotyping resolution obtained by cloning is identical to the resolution obtained by pyrosequencing.

Using this approach, we genotyped PBMC samples from 61 animals. We detected an average of 1836 total sequence reads per animal, representing an average of 10.7 distinct transcripts per animal. The *KIR *genotypes of three half-siblings are shown in Figure 5. For each animal, the distinct *KIRs *detected are shown, along with the relative frequency of each *KIR*. In most cases, each sequence read unambiguously matched a single *KIR *allele, providing typing resolution to the allele level. When reads were identical to more than one known *KIR*, the result is presented either as a set of alleles (ie. Mamu-KIR3DL10*001/004, meaning the animal is positive for either Mamu-KIR3DL10*001 or *004) or as positive for a gene (ie. *Mamu-KIR1Dg*, meaning the animal has an allele of this gene). The latter is comparable to the level of resolution commonly provided by sequence specific PCR. To validate this technique, we compared the results of pyrosequencing with cloning data (Figure 5 and Additional file 1, Table S3). Because pyrosequencing examines hundreds or thousands of clones, as opposed to the tens of clones generally examined in conventional cloning, the former is able to detect more transcripts per animal, providing a more comprehensive genotype. An example is seen in r95061 with *Mamu-KIR3DS06*, which was detected by pyrosequencing, but was not detected by cloning. While the 623bp amplicon used in pyrosequencing was frequently able to provide allele-level typing, there are examples for which full-length cloning provided higher resolution typing, as we observe for KIR3DL01*001 and KIR3DL10*004 in r95061 (Figure 5).

A key advantage of pyrosequencing is that it provides estimates of the relative transcript frequency for each KIR. This is a dimension not usually captured by genotyping techniques. The three animals in Figure 5 share a *KIR *haplotype containing *Mamu-KIR1D*, Mamu-KIR3DL1*001, Mamu-KIR3DL10*004 and Mamu-KIR3DS04*002 (Figure 5, striped bars). *KIR *expression is influenced by the complete *KIR *and *MHC *genotype of the subject [10,17,39]. Work in humans has shown that *KIR *genotype is only loosely predictive of *KIR *RNA expression [40]. While the animals in Figure 5 share one *KIR *haplotype, their second *KIR *haplotypes are distinct, and none are *MHC *identical (data not shown). We observe considerable differences in the expression level of the *KIRs *on the shared haplotype, which is likely attributable to differences in *KIR/MHC *genotype. While these findings are not unexpected given the complex regulation of *KIR *expression, they do underscore the need to examine factors beyond the simple presence or absence of a given *KIR*.

**Figure 6:**
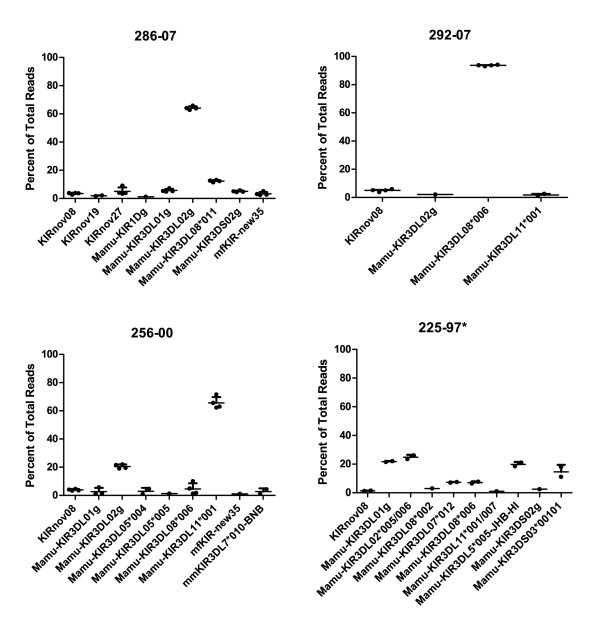
**Pyrosequencing produces reproducible frequency estimates for *KIR *transcripts**. Pyrosequencing results from four animals are shown. Per animal, two independent cell preparations were performed, and two independent PCRs were performed per cell preparation. For each replicate, the expression of each KIR is graphed as a percentage of total reads. Bars represent the average and standard deviation among replicates. For animal 225-97 (asterisk), only one cell pellet was available, so only two data points are shown.

To validate this technique, we examined the reproducibility of KIR expression levels. We performed two independent NK cell preparations from four animals, and two independent PCRs from each cell preparation. The resulting PCR amplicons were pyrosequenced. A graph of the relative expression level of each *KIR *is shown in Figure 6, with the full data in Additional file 1, Table S4. The frequency of each *KIR *was highly reproducible between sample preparations and PCR replicates. In every instance for which the average expression of a *KIR *was greater than 3% of total reads, that *KIR *transcript was detected in all replicates, demonstrating high sensitivity. It should be noted that transcripts present in less than 3% of total transcripts were missed in some reactions. There was also greater variability in detected expression levels among lower frequency transcripts. Of interest, in three of four animals, a single *KIR *transcript was dominant, representing greater than 60% of reads. Animal 225-97 was an exception, where four distinct *KIRs *comprise between 15-20% of the population each.

**Figure 7:**
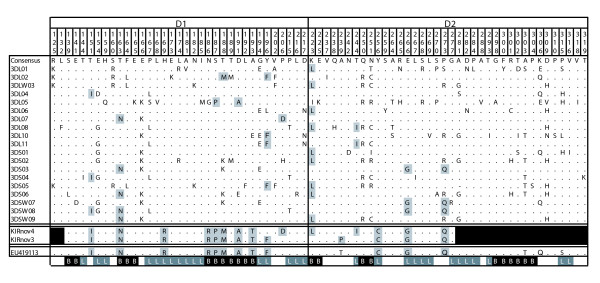
**Novel rhesus macaque *KIR *sequences share homology with cynomolgus macaque *KIRs***. Amino acid differences between the consensus sequences for the lineage II *Mamu-KIR *genes, *KIRnov03*, *KIRnov04 *and the Mafa-KIR sequence EU419113 are shown. The amino-acid position is indicated above the amino-acid sequences. The positions in the novel sequences that vary from the consensus are highlighted. Residues predicted to be involved in MHC-binding or alpha-helix loops are indicated below the sequences with B or L respectively. Black shading indicates residues for which no sequence coverage was available.

Another advantage of sequence-based typing, as opposed to techniques such as sequence-specific PCR, is that it is not limited to previously described alleles. From this data we were able to characterize an additional 44 novel partial-length rhesus macaque *KIR *sequences, 32 of which were found in multiple animals. As was the case with our novel full-length sequences, most novel *KIR *sequences showed significant sequence homology to the phylogenetic groups defined in Figure 1 (Additional file 1, Table S5), further supporting these putative gene groupings. While the majority of novel sequences were similar to these groups, we also identified two novel sequences, *KIRnov03 *and *KIRnov04*, that are similar to *Mamu-KIR3DS03*, but with a distinctive sequence motif in D1 (Figure 7). These sequences are of interest because they share this motif with a sequence previously found only in cynomolgus macaques (*Macaca fascicularis*) (EU419113). The unique residues for these sequences (186-193) correspond to predicted MHC class I binding sites [41]. These sequences may represent an additional macaque KIR gene, or a lineage within *KIR3DS03*.

### Population survey of KIR gene frequency

**Figure 8:**
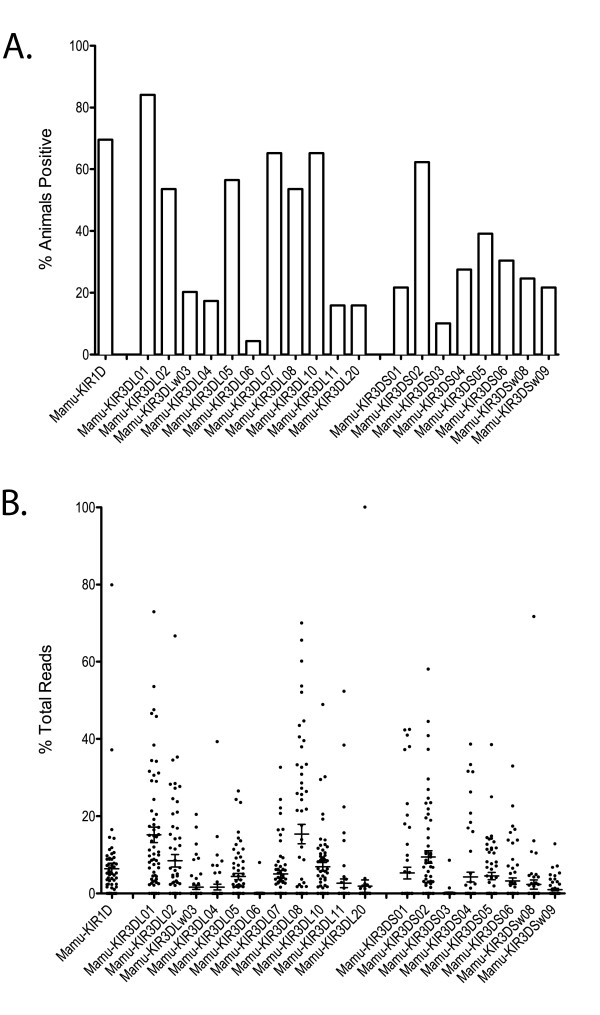
**Frequency and relative expression of *KIR *genes in the rhesus macaque cohort**. A) Y-axis indicates the percentage of animals within the cohort (n = 69) that express the indicated *KIR *gene. Genes not listed were not present in any animal within our cohort. *Mamu-KIR2DL04 *was excluded since it is not amplified by our pyrosequencing amplicon. B) Graph illustrates the percent of total pyrosequencing reads per animal (n = 61) for each *KIR *gene. Averages and SEM are represented by error bars. Each animal included had at least 100 sequencing reads. Genotyping results representing less than 1% of total reads in an animal were excluded to mitigate the influence of PCR artifacts. Ambiguous reads matching more than one *KIR *gene and splice variants were also excluded.

Obtaining comprehensive *KIR *genotypes from this large cohort allowed us to examine the relative frequency of each *KIR *gene at the population level (Figure 8A). We combined the pyrosequencing data with full-length cloning data to form a 69-animal cohort. While no *KIR *gene was present in every animal, *Mamu-KIR3DL01 *was present in approximately 84% of the cohort, making it the closest approximation to a framework gene in this population. While *Mamu-KIR3DL20, -KIR3DL11*, and -*KIR3DSW08 *have been proposed as framework genes for this species, each was present in less than 25% of this cohort [27]. It should be noted that our approach will identify transcribed KIRs only, while Kruse et al. employed sequence-specific PCR from genomic DNA. Our previous work in cynomolgus macaques suggests that *Mafa-KIR3DL20 *is commonly found as a pseudogene [28], which would be detected from gDNA, but not mRNA. It is possible some rhesus macaque haplotypes also have Mamu-KIR3DL20 as a pseudogene. While differences in technique may explain some discrepancies, it is also possible that the distinct breeding populations of rhesus macaques have distinctive genetic compositions. *Mamu- KIR2DL04 *has also been suggested as a framework gene in macaques [27], although *-KIR2DL04 *was only identified in 2 of 8 published cynomolgus macaque haplotypes [28]. As noted previously, the primers used in this study select against *Mamu-KIR2DL04 *transcripts, so we could not determine the presence or absence of this gene.

Using the pyrosequencing data generated from our cohort of 61 animals, we compared the relative contribution of each *KIR *gene to the total *KIR *transcripts identified in each animal (Figure 8B). Although pyrosequencing provides information about the *KIR *allele, genotyping results were condensed to the gene level for this comparison. The error bars representing the mean and SEM for each gene demonstrate highly variable expression levels for most genes between subjects, with a few *KIRs *consistently expressed at either high (i.e. *Mamu-KIR3DL08*) or low (i.e. *Mamu-KIR3DSw09*) levels. As observed in Figure 5, the expression level of a given *KIR *gene can differ even between genetically similar subjects and is likely influenced by the complete *KIR/MHC *genotype of that subject. While it would be interesting to examine the relative expression of each *KIR *in the context of *MHC *genotype, macaques can express up to 20 distinct *MHC *class I transcripts and only a handful of macaque KIR/MHC binding partners have been identified [42,43]. Therefore this analysis is not practical until physiological macaque KIR/MHC interactions are better understood.

### Rhesus macaque KIR haplotypes

**Figure 9:**
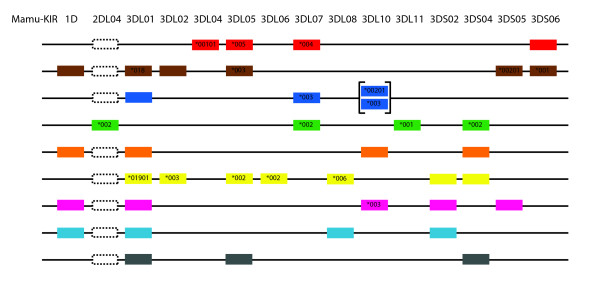
**Novel rhesus macaque *KIR *haplotypes**. *KIR *genes are indicated along the top axis. The identity of the allele is indicated within the schematic boxes if it was determined. Because data were generated from cDNA expression, only expressed *KIRs *are shown, and the physical map of gene order is arbitrary. Brackets indicate gene duplication. Since *Mamu-KIR2DL04 *cannot be amplified by our pyrosequencing amplicon, dotted boxes indicate haplotypes in which a false negative typing for this locus is possible.

While not all members of this cohort had pedigree information available, there were five distinct pedigreed families within the cohort for which segregation analysis was possible, with a total of 46 animals. By performing segregation analysis using genotypes obtained from cloning and pyrosequencing data, we were able to infer the *KIR *gene content of 9 haplotypes (Figure 9). Because these haplotypes were defined using segregation analyses, the linear order of KIR genes cannot be conclusively determined and the physical map of gene order shown in Figure 9 is arbitrary. It should be noted that this analysis allows only for definition of the minimum number of genes on a given haplotype, as heterozygosity versus homozygosity is sometimes not possible to rigorously determine, and low-level transcripts may not be detected in all animals with a given haplotype [30]. Among our 9 haplotypes, each was unique as compared to previously published rhesus macaque *KIR *haplotypes, with an average of 4.6 genes present per haplotype (Range: 3-7) [25,27,28,30]. In accordance with previous studies, we noted one incidence of a duplicated *KIR3DL *gene (*Mamu-KIR3DL10*) [27,30]. As described above, there is a notable absence of framework genes among these haplotypes. *Mamu-KIR3DL01 *represents the most common *KIR *in our cohort, yet it is present in only 7 of the 9 haplotypes.

## Discussion

Killer immunoglobulin-like receptor signaling is implicated in the immune response to numerous human pathogens, yet elucidating the role of KIR mediated signaling in disease pathogenesis is difficult in human subjects. The results of this study advance our understanding of macaque *KIRs*, enabling the study of KIR activity in this important non-human primate model for many human diseases. This study identified 47 novel full-length rhesus macaque *KIRs*, a substantial increase in the number of published sequences. Combined with previously published data, our results confirm the existence of 22 common macaque *KIR *genes, with extensive allelic variation within each gene (Guethlein et al, in preparation). This model of macaque *KIR *genetics provides an essential framework within which to describe and characterize macaque *KIRs*. In addition, among the novel sequences found using pyrosequencing, two sequences provide evidence for the existence of either a divergent lineage of *Mamu-KIR3DS03 *or an additional gene. While no *KIR *gene was present in all subjects, *Mamu-KIR3DL01 *was expressed in 84% of subjects and 8 genes were present in >50% of the cohort. This frequency information may be useful to prioritize *KIRs *for further functional characterization or the design of cohorts. The comparative lack of expressed framework genes is a distinction from human *KIR *haplotypes, which may be related to differences in *MHC-I *genetics. Humans express a maximum of six distinct HLA molecules, from 3 genomic loci. Macaques lack *MHC-I C*, but can express as many as 20 distinct *MHC-I A/B *alleles [44]. The expansion of macaque lineage II *KIR3D *loci, with a larger number of loci and few dominant genes, likely mirrors the expansion of macaque *MHC-I A/B*.

We also present a novel pyrosequencing-based approach for *KIR *genotyping. While this technique was developed in macaques, it could easily be applied to other species, including humans. The ability to characterize the *KIR *genotype of individual subjects is a basic requirement for the study of KIR function; however, the *KIR *region presents many challenges for genotyping. The approach we present comprehensively identifies the expressed *KIR *transcripts and provides a semi-quantitative measure of the relative expression level for each *KIR*. Our data demonstrate that even subjects sharing a *KIR *haplotype can have widely different expression levels of these shared *KIRs*. NK cells undergo complex regulation of *KIR *expression [10,17]. NK clones expressing fewer distinct *KIRs *can have a reduced activation threshold under certain conditions, resulting in enhanced NK cell activity [45]. The relative expression of each *KIR *therefore provides potentially important functional information.

While this and other recent studies have advanced our understanding of the genetic organization and diversity of macaque *KIRs*, KIR activity must be understood in the context of *MHC *genotype. The limited knowledge of functional KIR/MHC binding partners remains a key obstacle. Recent work has identified multiple macaque KIR/MHC-I interactions, including allotypes of Mamu-KIR3DL05, -KIR3DLW03, -KIR3DL11 and -KIR3DS05 [43,46]. Preliminary evidence suggests the residues of the Bw4/Bw6 motifs influence KIR/MHC binding in macaques, although the important residues differ from those in humans. Expanding the number of identified KIR/MHC partners and further defining the motifs necessary for macaque KIR/MHC interaction remains essential for the advancement of the field.

## Conclusions

The data and techniques presented here have implications for the study of many diseases using rhesus macaques as a model system and for the KIR field as a whole. The ability to study KIRs in a nonhuman primate model creates an opportunity for significant advancement in our understanding of KIRs in human disease. The novel high-throughput *KIR *genotyping method we developed has implications for many species, including potential application in humans, and the approach could be adapted for other polymorphic immune loci.

## Authors' contributions

AJM was involved in data collection, data analysis and writing the manuscript. LAG and PP performed data analysis and assisted with manuscript writing. RKR performed NK cell sample preparations and was involved in the experimental design. KWB performed statistical analysis of pyrosequencing data. RPJ and DHO provided grant support and were involved in the experimental design. BNB was involved in experimental design, data collection, data analysis and manuscript writing. All authors have read and approve the final manuscript.

## Endnotes

^1 ^A note on nomenclature: KIR nomenclature reflects both the domain structure and gene designation of the sequence. For example, an allele named "KIR3DL01*001" denotes a KIR with 3 extracellular immunoglobulin-like domains (3D). The "L" indicates that this allele's protein product has a long, inhibitory cytoplasmic tail, while an "S" would instead indicate that the protein product had a short, activating cytoplasmic tail. The next two digits refer to the gene, in this case KIR3DL01. Finally, the digits after the asterisk, "001" in this example, refer to the specific allele.

^2 ^Human KIRs are divided into four lineages. Lineage I includes KIR2D genes having a D0 (immunoglobulin-like domain 0) plus D2 configuration such as KIR2DL4 and KIR2DL5; lineage II includes the KIR3DL genes that bind MHC-A and -B epitopes; lineage III includes KIR3D or KIR2D genes with a D1 plus D2 domain configuration that bind MHC-C epitopes, and lineage V is represented by KIR3DL3. Lineage IV, which included Mamu-KIR3DL01 and Mamu-KIR3DL10, was originally described in Rajalingam, et al. [37]. Further work has instead grouped these KIRs into lineage II and removed lineage IV [47].

## Supplementary Material

Additional file 1 **Figure S1. Alignment of all published rhesus macaque lineage II KIR sequences**. An alignment was generated from all previously published rhesus macaque KIRs using predicted amino acid sequences. Sequences identified in this publication are highlighted. KIRs are grouped by gene, and a consensus sequence is included for each gene. An asterisk after the accession number indicates this sequence was published multiple times and only one of the accession numbers is given. **Table S1. Genbank accession numbers for novel full-length rhesus macaque *KIR *sequences**. Sequences have been assigned official names through the Immuno Polymorphism Database. **Table S2. PCR primers used for cDNA-PCR pyrosequencing. Table S3. Comparison of pyrosequencing and cloning results**. For each animal, detected *KIR *alleles are shown, along with their relative frequency detected by pyrosequencing, expressed as a percent of total 454 reads. The column on the right indicates the result of conventional cloning. A plus sign indicates that the detected resolution matches the corresponding pyrosequencing result. If cloning resulted in a different resolution, the corresponding allele name is shown. **Table S4. Reproducibility of allele frequency estimates**. Pyrosequencing data from four animals are shown. Two independent NK cell isolations were performed per animal, and two independent PCRs were performed per cell preparation, with the exception of 225-07, for which only one cell pellet was available. The resulting PCR amplicons were pyrosequenced. The total number of reads is shown for each reaction. *KIRs *detected are shown, expressed as a percent of total reads. **Table S5. GenBank accession numbers for novel partial length rhesus macaque *KIR *sequences identified by pyrosequencing**. Sequences have been assigned sequential, unofficial names. For each sequence, the *KIR *allele or gene to which it bears greatest similarity is indicated. The Total Ids column indicates the number of distinct animals in which that sequence was observed. Abbreviations: u: unique; gc: gene conversion; r: recombination; sv: splice variant. Click here for file

## References

[B1] Lanier LL Evolutionary struggles between NK cells and viruses Nat Rev Immunol 2008 8 4 259 268 10.1038/nri2276 18340344 PMC2584366

[B2] Gardiner CM Killer cell immunoglobulin-like receptors on NK cells: the how, where and why Int J Immunogenet 2008 35 1 1 8 18093180 10.1111/j.1744-313X.2007.00739.x

[B3] Parham P MHC class I molecules and KIRs in human history, health and survival Nat Rev Immunol 2005 5 3 201 214 10.1038/nri1570 15719024

[B4] Beck JC Wagner JE DeFor TE Brunstein CG Schleiss MR Young JA Weisdorf DH Cooley S Miller JS Verneris MR Impact of cytomegalovirus (CMV) reactivation after umbilical cord blood transplantation Biol Blood Marrow Transplant 2010 16 2 215 222 10.1016/j.bbmt.2009.09.019 19786112 PMC2819578

[B5] Miller JS Cooley S Parham P Farag SS Verneris MR McQueen KL Guethlein LA Trachtenberg EA Haagenson M Horowitz MM Missing KIR ligands are associated with less relapse and increased graft-versus-host disease (GVHD) following unrelated donor allogeneic HCT Blood 2007 109 11 5058 5061 10.1182/blood-2007-01-065383 17317850 PMC1885526

[B6] Khakoo S Thio C Martin M Brooks C Gao X Astemborski J Cheng J Goedert J Vlahov D Hilgartner M HLA and NK cell inhibitory receptor genes in resolving hepatitis C virus infection Science 2004 305 5685 872 874 10.1126/science.1097670 15297676

[B7] Bonagura VR Du Z Ashouri E Luo L Hatam LJ DeVoti JA Rosenthal DW Steinberg BM Abramson AL Gjertson DW Activating killer cell immunoglobulin-like receptors 3DS1 and 2DS1 protect against developing the severe form of recurrent respiratory papillomatosis Hum Immunol 2010 71 2 212 219 10.1016/j.humimm.2009.10.009 19861144 PMC2815039

[B8] Martin M Qi Y Gao X Yamada E Martin J Pereyra F Colombo S Brown E Shupert W Phair J Innate partnership of HLA-B and KIR3DL1 subtypes against HIV-1 Nat Genet 2007 39 6 733 740 10.1038/ng2035 17496894 PMC4135476

[B9] Martin M Gao X Lee J Nelson G Detels R Goedert J Buchbinder S Hoots K Vlahov D Trowsdale J Epistatic interaction between KIR3DS1 and HLA-B delays the progression to AIDS Nat Genet 2002 31 4 429 434 12134147 10.1038/ng934

[B10] Kim S Poursine-Laurent J Truscott S Lybarger L Song Y Yang L French A Sunwoo J Lemieux S Hansen T Licensing of natural killer cells by host major histocompatibility complex class I molecules Nature 2005 436 7051 709 713 10.1038/nature03847 16079848

[B11] Moretta L Moretta A Killer immunoglobulin-like receptors Curr Opin Immunol 2004 16 5 626 633 10.1016/j.coi.2004.07.010 15342010

[B12] Wilson M Torkar M Haude A Milne S Jones T Sheer D Beck S Trowsdale J Plasticity in the organization and sequences of human KIR/ILT gene families Proc Natl Acad Sci USA 2000 97 9 4778 4783 10.1073/pnas.080588597 10781084 PMC18309

[B13] Hsu K Chida S Geraghty D Dupont B The killer cell immunoglobulin-like receptor (KIR) genomic region: gene-order, haplotypes and allelic polymorphism Immunol Rev 2002 190 40 52 10.1034/j.1600-065X.2002.19004.x 12493005

[B14] Witt C Dewing C Sayer D Uhrberg M Parham P Christiansen F Population frequencies and putative haplotypes of the killer cell immunoglobulin-like receptor sequences and evidence for recombination Transplantation 1999 68 11 1784 1789 10.1097/00007890-199912150-00024 10609957

[B15] Vilches C Parham P KIR: diverse, rapidly evolving receptors of innate and adaptive immunity Annu Rev Immunol 2002 20 217 251 10.1146/annurev.immunol.20.092501.134942 11861603

[B16] Yamada S Hatta M Staker BL Watanabe S Imai M Shinya K Sakai-Tagawa Y Ito M Ozawa M Watanabe T Biological and structural characterization of a host-adapting amino acid in influenza virus PLoS Pathog 2010 6 8 10.1371/journal.ppat.1001034 PMC2916879 20700447

[B17] Shilling HG Young N Guethlein LA Cheng NW Gardiner CM Tyan D Parham P Genetic control of human NK cell repertoire J Immunol 2002 169 1 239 247 12077250 10.4049/jimmunol.169.1.239

[B18] Hansen D D'Ombrain M Schofield L The role of leukocytes bearing Natural Killer Complex receptors and Killer Immunoglobulin-like Receptors in the immunology of malaria Curr Opin Immunol 2007 19 4 416 423 10.1016/j.coi.2007.07.011 17702559

[B19] Alter G Martin MP Teigen N Carr WH Suscovich TJ Schneidewind A Streeck H Waring M Meier A Brander C Differential natural killer cell-mediated inhibition of HIV-1 replication based on distinct KIR/HLA subtypes J Exp Med 2007 204 12 3027 3036 10.1084/jem.20070695 18025129 PMC2118524

[B20] Bontrop RE Watkins DI MHC polymorphism: AIDS susceptibility in non-human primates Trends Immunol 2005 26 4 227 233 10.1016/j.it.2005.02.003 15797514

[B21] O'Connor D Allen T Watkins D Cytotoxic T-lymphocyte escape monitoring in simian immunodeficiency virus vaccine challenge studies DNA Cell Biol 2002 21 9 659 664 10.1089/104454902760330192 12396608

[B22] Loffredo J Burwitz B Rakasz E Spencer S Stephany J Vela J Martin S Reed J Piaskowski S Furlott J The antiviral efficacy of simian immunodeficiency virus-specific CD8+ T cells is unrelated to epitope specificity and is abrogated by viral escape J Virol 2007 81 6 2624 2634 10.1128/JVI.01912-06 17192314 PMC1866004

[B23] Hershberger K Shyam R Miura A Letvin N Diversity of the killer cell Ig-like receptors of rhesus monkeys J Immunol 2001 166 7 4380 4390 11254692 10.4049/jimmunol.166.7.4380

[B24] Grendell RL Hughes AL Golos TG Cloning of rhesus monkey killer-cell Ig-like receptors (KIRs) from early pregnancy decidua Tissue Antigens 2001 58 5 329 334 10.1034/j.1399-0039.2001.580507.x 11844144

[B25] Sambrook J Bashirova A Palmer S Sims S Trowsdale J Abi-Rached L Parham P Carrington M Beck S Single haplotype analysis demonstrates rapid evolution of the killer immunoglobulin-like receptor (KIR) loci in primates Genome Res 2005 15 1 25 35 10.1101/gr.2381205 15632087 PMC540275

[B26] Blokhuis JH van der Wiel MK Doxiadis GG Bontrop RE Evidence for balancing selection acting on KIR2DL4 genotypes in rhesus macaques of Indian origin Immunogenetics 2009 61 7 503 512 10.1007/s00251-009-0379-6 19506858

[B27] Kruse PH Rosner C Walter L Characterization of rhesus macaque KIR genotypes and haplotypes Immunogenetics 2010 62 5 281 293 10.1007/s00251-010-0433-4 20195593

[B28] Bimber BN Moreland AJ Wiseman RW Hughes AL O'Connor DH Complete characterization of killer Ig-like receptor (KIR) haplotypes in Mauritian cynomolgus macaques: novel insights into nonhuman primate KIR gene content and organization J Immunol 2008 181 9 6301 6308 18941221 10.4049/jimmunol.181.9.6301 PMC2832209

[B29] Robinson J Marsh SG IPD: the Immuno Polymorphism Database Methods Mol Biol 2007 409 61 74 10.1007/978-1-60327-118-9_4 18449992

[B30] Blokhuis JH van der Wiel MK Doxiadis GG Bontrop RE The mosaic of KIR haplotypes in rhesus macaques Immunogenetics 2010 62 5 295 306 10.1007/s00251-010-0434-3 20204612 PMC2858804

[B31] Thompson JD Higgins DG Gibson TJ CLUSTAL W: improving the sensitivity of progressive multiple sequence alignment through sequence weighting, position-specific gap penalties and weight matrix choice Nucleic Acids Res 1994 22 22 4673 4680 10.1093/nar/22.22.4673 7984417 PMC308517

[B32] Blokhuis JH Doxiadis GG Bontrop RE A splice site mutation converts an inhibitory killer cell Ig-like receptor into an activating one Mol Immunol 2009 46 4 640 648 10.1016/j.molimm.2008.08.270 19019442

[B33] Li H Handsaker B Wysoker A Fennell T Ruan J Homer N Marth G Abecasis G Durbin R The Sequence Alignment/Map format and SAMtools Bioinformatics 2009 25 16 2078 2079 10.1093/bioinformatics/btp352 19505943 PMC2723002

[B34] Stajich JE Block D Boulez K Brenner SE Chervitz SA Dagdigian C Fuellen G Gilbert JG Korf I Lapp H The Bioperl toolkit: Perl modules for the life sciences Genome Res 2002 12 10 1611 1618 10.1101/gr.361602 12368254 PMC187536

[B35] Robinson J Mistry K McWilliam H Lopez R Marsh SG IPD--the Immuno Polymorphism Database Nucleic Acids Res 2010 38 Database D863 869 10.1093/nar/gkp879 PMC2808958 19875415

[B36] Hershberger KL Kurian J Korber BT Letvin NL Killer cell immunoglobulin-like receptors (KIR) of the African-origin sabaeus monkey: evidence for recombination events in the evolution of KIR Eur J Immunol 2005 35 3 922 935 10.1002/eji.200425408 15714591

[B37] Rajalingam R Parham P Abi-Rached L Domain shuffling has been the main mechanism forming new hominoid killer cell Ig-like receptors J Immunol 2004 172 1 356 369 14688344 10.4049/jimmunol.172.1.356

[B38] Bashirova AA Martin MP McVicar DW Carrington M The killer immunoglobulin-like receptor gene cluster: tuning the genome for defense Annu Rev Genomics Hum Genet 2006 7 277 300 10.1146/annurev.genom.7.080505.115726 16824023

[B39] Kim S Sunwoo JB Yang L Choi T Song YJ French AR Vlahiotis A Piccirillo JF Cella M Colonna M HLA alleles determine differences in human natural killer cell responsiveness and potency Proc Natl Acad Sci USA 2008 105 8 3053 3058 10.1073/pnas.0712229105 18287063 PMC2268583

[B40] McErlean C Gonzalez AA Cunningham R Meenagh A Shovlin T Middleton D Differential RNA expression of KIR alleles Immunogenetics 2010 62 7 431 440 10.1007/s00251-010-0449-9 20454893

[B41] Sharma D Bastard K Guethlein LA Norman PJ Yawata N Yawata M Pando M Thananchai H Dong T Rowland-Jones S Dimorphic motifs in D0 and D1+D2 domains of killer cell Ig-like receptor 3DL1 combine to form receptors with high, moderate, and no avidity for the complex of a peptide derived from HIV and HLA-A*2402 J Immunol 2009 183 7 4569 4582 10.4049/jimmunol.0901734 19752231 PMC2827337

[B42] Wiseman RW Karl JA Bimber BN O'Leary CE Lank SM Tuscher JJ Detmer AM Bouffard P Levenkova N Turcotte CL Major histocompatibility complex genotyping with massively parallel pyrosequencing Nat Med 2009 15 11 1322 1326 10.1038/nm.2038 19820716 PMC2824247

[B43] Rosner C Kruse PH Hermes M Otto N Walter L Rhesus Macaque Inhibitory and Activating KIR3D Interact with Mamu-A-Encoded Ligands J Immunol 2011 186 4 2156 2163 10.4049/jimmunol.1002634 21257962

[B44] Budde ML Lhost JJ Burwitz BJ Becker EA Burns CM O'Connor SL Karl JA Wiseman RW Bimber BN Zhang GL Transcriptionally Abundant Major Histocompatibility Complex Class I Alleles are Fundamental to Non-Human Primate SIV-specific CD8+ T Cell Responses J Virol 2011 10.1128/JVI.02355-10 PMC3067831 21270169

[B45] Carrington M Martin MP van Bergen J KIR-HLA intercourse in HIV disease Trends Microbiol 2008 16 12 620 627 10.1016/j.tim.2008.09.002 18976921 PMC3463869

[B46] Colantonio AD Bimber BN Neidermyer WJ Reeves RK Alter G Altfeld M Johnson RP Carrington M O'Connor DH Evans DT KIR Polymorphisms Modulate Peptide-Dependent Binding to an MHC Class I Ligand with a Bw6 Motif PLoS Pathog 2011 7 3 e1001316 10.1371/journal.ppat.1001316 21423672 PMC3053351

[B47] Guethlein LA Older Aguilar AM Abi-Rached L Parham P Evolution of killer cell Ig-like receptor (KIR) genes: definition of an orangutan KIR haplotype reveals expansion of lineage III KIR associated with the emergence of MHC-C J Immunol 2007 179 1 491 504 17579070 10.4049/jimmunol.179.1.491

