# Supplementary material for: Characterization of killer immunoglobulin-like receptor genetics and comprehensive genotyping by pyrosequencing in rhesus macaques
Source: BMC Genomics. 2011 Jun 7;12:295. doi: 10.1186/1471-2164-12-295 (PMC3125267; doi:10.1186/1471-2164-12-295)
Supplement: Additional file 1 — Figure S1. Alignment of all published rhesus macaque lineage II KIR sequences. An alignment was generated from all previously published rhesus macaque KIRs using predicted amino acid sequences. Sequences identified in this publication are highlighted. KIRs are grouped by gene, and a consensus sequence is included for each gene. An asterisk after the accession number indicates this sequence was published multiple times and only one of the accession numbers is given. Table S1. Genbank accession numbers for novel full-length rhesus macaque KIR sequences. Sequences have been assigned official names through the Immuno Polymorphism Database. Table S2. PCR primers used for cDNA-PCR pyrosequencing. Table S3. Comparison of pyrosequencing and cloning results. For each animal, detected KIR alleles are shown, along with their relative frequency detected by pyrosequencing, expressed as a percent of total 454 reads. The column on the right indicates the result of conventional cloning. A plus sign indicates that the detected resolution matches the corresponding pyrosequencing result. If cloning resulted in a different resolution, the corresponding allele name is shown. Table S4. Reproducibility of allele frequency estimates. Pyrosequencing data from four animals are shown. Two independent NK cell isolations were performed per animal, and two independent PCRs were performed per cell preparation, with the exception of 225-07, for which only one cell pellet was available. The resulting PCR amplicons were pyrosequenced. The total number of reads is shown for each reaction. KIRs detected are shown, expressed as a percent of total reads. Table S5. GenBank accession numbers for novel partial length rhesus macaque KIR sequences identified by pyrosequencing. Sequences have been assigned sequential, unofficial names. For each sequence, the KIR allele or gene to which it bears greatest similarity is indicated. The Total Ids column indicates the number of distinct animals in which that sequen [file 1471-2164-12-295-S1.PDF]

Table S1. Genbank accession numbers for novel full length rhesus macaque *KIR* sequences. Sequences have been assigned official names through the Immuno Polymorphism Database.

Allele	Genbank Accession
Mamu-KIR3DL01*013	EU419034
Mamu-KIR3DL01*014	EU419035
Mamu-KIR3DL01*015	EU419036
Mamu-KIR3DL01*016	EU419037
Mamu-KIR3DL01*017	EU419044
Mamu-KIR3DL01*018	EU419046
Mamu-KIR3DL01*01901	EU419032
Mamu-KIR3DL01*020	EU688987
Mamu-KIR3DL02*003	EU419050
Mamu-KIR3DL02*00401	EU419052
Mamu-KIR3DL02*00402	EU419053
Mamu-KIR3DL02*005	EU419054
Mamu-KIR3DL02*006	EU688989
Mamu-KIR3DL04*00101	EU419040
Mamu-KIR3DL04*002	EU419042
Mamu-KIR3DL05*001	EU419045
Mamu-KIR3DL05*002	EU419061
Mamu-KIR3DL05*003	EU419062
Mamu-KIR3DL05*004	EU419066
Mamu-KIR3DL05*005	EU419069
Mamu-KIR3DL05*00601	EU688991
Mamu-KIR3DL06*002	EU419056
Mamu-KIR3DL07*003	EU419057
Mamu-KIR3DL07*004	EU419060
Mamu-KIR3DL07*005	EU419063
Mamu-KIR3DL07*006	EU419064
Mamu-KIR3DL07*007	EU419065
Mamu-KIR3DL07*008	EU419068
Mamu-KIR3DL07*00901	EU688990
Mamu-KIR3DL07*010	EU688992
Mamu-KIR3DL08*00102	EU419071
Mamu-KIR3DL08*006	EU419070
Mamu-KIR3DL10*003	EU419038
Mamu-KIR3DL10*004	EU419039
Mamu-KIR3DL20*005	EU419047
Mamu-KIR3DL20*006	EU419048
Mamu-KIR3DL20*007	EU419049

Mamu-KIR3DLW03*001	EU419051
Mamu-KIR3DLW03*002	EU419055
Mamu-KIR3DLW03*003	EU419031
Mamu-KIR3DS02*005	EU419027
Mamu-KIR3DS04*002	EU419028
Mamu-KIR3DS04*003	EU419029
Mamu-KIR3DS05*001	EU419024
Mamu-KIR3DS06*00201	EU688985
Mamu-KIR3DSW09*001	EU419030
Mamu-KIR3DSW09*002	EU688986

Table S2. PCR primers used for cDNA-PCR pyrosequencing.

Oligonucleotide Name	Oligonucleotide Sequence
KIR-405-F_MID01	CGTATCGCCTCCCTCGCGCCATCAGACGAGTGCGTAGGTCCCCTGGTGAAATCAG
KIR-405-F_MID02	CGTATCGCCTCCCTCGCGCCATCAGACGCTCGACAAGGTCCCCTGGTGAAATCAG
KIR-405-F_MID03	CGTATCGCCTCCCTCGCGCCATCAGAGACGCACTCAGGTCCCCTGGTGAAATCAG
KIR-405-F_MID04	CGTATCGCCTCCCTCGCGCCATCAGAGCACTGTAGAGGTCCCCTGGTGAAATCAG
KIR-405-F_MID05	CGTATCGCCTCCCTCGCGCCATCAGATCAGACACGAGGTCCCCTGGTGAAATCAG
KIR-405-F_MID06	CGTATCGCCTCCCTCGCGCCATCAGATATCGCGAGAGGTCCCCTGGTGAAATCAG
KIR-405-F_MID07	CGTATCGCCTCCCTCGCGCCATCAGCGTGTCTCTAAGGTCCCCTGGTGAAATCAG
KIR-405-F_MID08	CGTATCGCCTCCCTCGCGCCATCAGCTCGCGTGTCAGGTCCCCTGGTGAAATCAG
KIR-405-F_MID09	CGTATCGCCTCCCTCGCGCCATCAGTAGTATCAGCAGGTCCCCTGGTGAAATCAG
KIR-405-F_MID10	CGTATCGCCTCCCTCGCGCCATCAGTCTCTATGCGAGGTCCCCTGGTGAAATCAG
KIR-405-F_MID11	CGTATCGCCTCCCTCGCGCCATCAGTGATACGTCTAGGTCCCCTGGTGAAATCAG
KIR-405-F_MID12	CGTATCGCCTCCCTCGCGCCATCAGTACTGAGCTAAGGTCCCCTGGTGAAATCAG
KIR-1004-R_MID01	CTATGCGCCTTGCCAGCCCGCTCAGACGAGTGCGTCTTGGTTCAGTGGGTGAAGG
KIR-1004-R_MID02	CTATGCGCCTTGCCAGCCCGCTCAGACGCTCGACACTTGGTTCAGTGGGTGAAGG
KIR-1004-R_MID03	CTATGCGCCTTGCCAGCCCGCTCAGAGACGCACTCCTTGGTTCAGTGGGTGAAGG
KIR-1004-R_MID04	CTATGCGCCTTGCCAGCCCGCTCAGAGCACTGTAGCTTGGTTCAGTGGGTGAAGG
KIR-1004-R_MID05	CTATGCGCCTTGCCAGCCCGCTCAGATCAGACACGCTTGGTTCAGTGGGTGAAGG
KIR-1004-R_MID06	CTATGCGCCTTGCCAGCCCGCTCAGATATCGCGAGCTTGGTTCAGTGGGTGAAGG
KIR-1004-R_MID07	CTATGCGCCTTGCCAGCCCGCTCAGCGTGTCTCTACTTGGTTCAGTGGGTGAAGG
KIR-1004-R_MID08	CTATGCGCCTTGCCAGCCCGCTCAGCTCGCGTGTCCTTGGTTCAGTGGGTGAAGG
KIR-1004-R_MID09	CTATGCGCCTTGCCAGCCCGCTCAGTAGTATCAGCCTTGGTTCAGTGGGTGAAGG
KIR-1004-R_MID10	CTATGCGCCTTGCCAGCCCGCTCAGTCTCTATGCGCTTGGTTCAGTGGGTGAAGG
KIR-1004-R_MID11	CTATGCGCCTTGCCAGCCCGCTCAGTGATACGTCTCTTGGTTCAGTGGGTGAAGG
KIR-1004-R_MID12	CTATGCGCCTTGCCAGCCCGCTCAGTACTGAGCTACTTGGTTCAGTGGGTGAAGG

Table S3. Comparison of pyrosequencing and cloning results. For each animal, detected *KIR* alleles are shown, along with their relative frequency detected by pyrosequencing, expressed as a percent of total 454 reads. The column on the right indicates the result of conventional cloning. A plus sign indicates that the detected resolution matches the corresponding pyrosequencing result. If cloning resulted in a different resolution, the corresponding allele name is shown.

r96112

Allele Name	% 454 Reads	Cloning
Mamu-KIR1D*001	0.5	+
Mamu-KIR3DL01*011/014/019	1.9	Mamu-KIR3DL01*019
Mamu-KIR3DL02g	4.4	Mamu-KIR3DL02*00401
Mamu-KIR3DL02*005/006	16.1	Mamu-KIR3DL02*005
Mamu-KIR3DL08*00102	47.9	+
Mamu-KIR3DL20*001	0.4	+
Mamu-KIR3DS01*001g	19.8	Mamu-KIR3DS01*00101
Mamu-KIR3DSW09*002	9.0	Not Detected
Total Sequences Examined:	916	135

r98016

Allele Name	% 454 Reads	Cloning
Mamu-KIR1Dg	55.6	Not Detected
Mamu-KIR3DL01*002/018	4.6	Not Detected
Mamu-KIR3DL07*003	0.8	+
Mamu-KIR3DL10*002	2.0	+
Mamu-KIR3DL10*003	2.1	+
Mamu-KIR3DS02g	30.3	Not Detected
Total Sequences Examined:	957	46

r97113

Allele Name	% 454 Reads	Cloning
Mamu-KIR1Dg	6.8	Not Detected
Mamu-KIR3DL01*013/015	18.7	**
	**	Mamu-KIR3DL01*013
	**	Mamu-KIR3DL01*015
Mamu-KIR3DL2*005	4.9	+
mmKIR3DL07*010-BNB	1.9	+
Mamu-KIR3DL08*006	32.3	Not Detected
Mamu-KIR3DL04*002	9.0	Not Detected
Mamu-KIR3DLW03*002	14.6	+
Mamu-KIR3DSW08*005	10.9	Not Detected
Total Sequences Examined:	773	112

Table S4. Reproducibility of allele frequency estimates. Pyrosequencing data from four animals are shown. Two independent NK cell pellets were prepared per animal, and two independent PCRs were performed per cell pellet, with the exception of 225-07, for which only one cell pellet was available. The resulting PCR amplicons were pyrosequenced. The total number of reads is shown for each reaction, with each *KIR* expressed as a percent of total reads. Standard deviation (S.D.) is shown to the right.

292-07

	Sample 1		Sample 2		
	PCR 1	PCR 2	PCR 1	PCR 2	S.D.
KIRnov08	5.9	3.8	5.1	5.0	0.87
Mamu-KIR3DL02g				2.0	0.00
Mamu-KIR3DL08*006	94.1	93.7	93.8	93.0	0.47
Mamu-KIR3DL11*001		2.5	1.1		0.99
**Total Reads:**	964	839	448	700	

256-00

	Sample 1		Sample 2		
	PCR 1	PCR 2	PCR 1	PCR 2	S.D.
KIRnov08	4.7	3.5	4.1	3.4	0.60
Mamu-KIR3DL01g	5.7	1.4		1.1	2.57
Mamu-KIR3DL02g	21.5	19.5	22.0	19.1	1.44
Mamu-KIR3DL05*004	1.1		4.6		2.47
Mamu-KIR3DL05*005			1.3		0.00
Mamu-KIR3DL08*006	1.8	10.1	1.3	4.9	4.04
Mamu-KIR3DL11*001	63.0	65.5	62.3	71.5	4.18
mfKIR-new35	1.1				0.00
mmKIR3DL7*010-BNB	1.1		4.3		2.26
**Total Reads:**	722	656	676	880	

286-07

	Sample 1		Sample 2		
	PCR 1	PCR 2	PCR 1	PCR 2	S.D.
KIRnov08	2.6	3.6	3.8	3.8	0.57
KIRnov19		2.1		1.6	0.35
KIRnov27	3.4	3.8	9.0	4.0	2.65
Mamu-KIR1Dg				1.2	0.00
Mamu-KIR3DL01g	4.9	7.2	5.1	5.3	1.06
Mamu-KIR3DL02g	65.8	63.8	62.8	64.0	1.25
Mamu-KIR3DL08*011	12.6	11.4	12.2	13.0	0.68
Mamu-KIR3DS02g	5.7	5.0	4.5	4.7	0.53
mfKIR-new35	5.1	3.0	2.6	2.4	1.24
**Total Reads:**	860	760	156	506	

225-97	Sample 1 PCR 1	Sample 1 PCR 2	S.D.
KIRnov08	1.5	1.3	0.14
Mamu-KIR3DL01g	22.0	21.5	0.35
Mamu-KIR3DL02*005/006	26.0	23.5	1.77
Mamu-KIR3DL08*002		3.0	0.00
Mamu-KIR3DL07*012	7.5	7.1	0.28
Mamu-KIR3DL08*006	6.5	7.8	0.92
Mamu-KIR3DL11*001/007		1.0	0.00
Mamu-KIR3DL5*005-JHB-HI	18.5	21.0	1.77
Mamu-KIR3DS02g		2.5	0.00
Mamu-KIR3DS03*00101	18.0	11.1	4.88
**Total Reads:**	200	395	

Table S5. GenBank accession numbers for novel partial length rhesus macaque KIR sequences identified by pyrosequencing. Sequences have been assigned sequential, unofficial names. For each sequence, the *KIR* allele or gene to which it bears greatest similarity is indicated. The Total Ids columnindicates the number of distinct animals in which that sequence was observed. Abbreviations: u: unique; gc: gene conversion; r: recombination; sv: splice variant.

Gene	Process	Sequence Name	Accession #	Closest Allele(s)	Total Ids
1D	u	KIRnov27	HQ124025	multiple 1D	63
	u	KIRnov26	HQ124024	multiple 1D	1
3DL02	u	KIRnov08	HQ124036	multiple 3DL02	99
3DLW03	u	KIRnov24	HQ124047	3DLW03*002	8
	gc,u	KIRnov15	HQ124042	multiple 3DLW03	6
3DL04	gc,u	KIRnov60	HQ124017	multiple 3DL04	2
3DL07	u	KIRnov34	HQ124049	3DL7*001	7
	u	KIRnov33	HQ124048	KIRnov34	6
	u	KIRnov20	HQ124045	3DL07*005	13
	u	KIRnov38	HQ124052	3DL07*001	4
	u	KIRnov54	HQ124054	KIRnov34	1
	u	KIRnov7	HQ124064	3DL07*008/010	1
	gc	KIRnov92	HQ124074	multiple 3DL07	1
	u	KIRnov81sv	HQ124068	3DL07*001	8
	sv,u	KIRnov88sv	HQ124072	3DL07*008/010	2

3DL08	gc	KIRnov59	HQ124057	3DL08*001	6
	r	KIRnov23	HQ124046	3DL08*001	15
	gc	KIRnov61	HQ124058	3DL08*002/009	11
	u	KIRnov72	HQ124065	3DL08*004/*007	15
	gc	KIRnov76	HQ124066	3DL08*001	3
3DL10	u,sv	KIRnov103sv	HQ124039	3DL10*002	1
	u	KIRnov2	HQ124044	3DL10*003	10
	u	KIRnov5	HQ124053	3DL10*002	71
3DL11	u,sv	KIRnov85sv	HQ124020	3DL11*001	3
	u	KIRnov10	HQ124037	3DL11*001	1
3DL20	u,sv	KIRnov91sv	HQ124073	multiple 3DL20	2
3DS01	gc	KIRnov55	HQ124086	3DS01*001/002	2
	gc,u	KIRnov35	HQ124050	multiple 3DS01	10
3DS02	gc,u	KIRnov09	HQ124076	3DS02*003/004/007/009/010	66
	gc,u	KIRnov11	HQ124077	multiple 3DS02	1
	gc,u	KIRnov12	HQ124078	multiple 3DS02	5
	u,sv	KIRnov93	HQ124094	multiple 3DS02	1
3DS03	gc	KIRnov19	HQ124043	3DS03*001	8
3DS03 like	u,gc	KIRnov3	HQ124083		4
	u,gc	KIRnov4	HQ124085		1
3DS04	gc	KIRnov18	HQ124079	multiple 3DS04	1
	u	KIRnov57	HQ124087	3DS04*002	1
	u,gc	KIRnov21	HQ124080	KIRnov57	3
	r	KIRnov65	HQ124089	multiple 3DS04	33
3DS06	gc	KIRnov71	HQ124091	3DS06*001/002/005	1
3DSW08	u,gc	KIRnov25	HQ124082	3DSW08*008	6
3DSW09	u,gc	KIRnov22	HQ124081	3DSW09*001	6
	gc	KIRnov62	HQ124018	3DSW09*001	2
	gc	KIRnov67	HQ124090	3DSW09*002/003	16

Figure S1. Alignment of all published rhesus macaque KIR sequences. An alignment was generated from all previously published rhesus macaque KIRs using predicted amino acid sequences. Sequences identified in this publication are highlighted. KIRs are ordered by gene, and a consensus sequence is included for each gene. An asterisk after the accession number indicates this sequence was published multiple times.

SIGNAL SEQUENCE

```
                        10        20
              ....|....|....|....|.
Consensus     MSLMVVSVACVGFFLVQRACP

AF334616      ...............I.TT..
AF334617      ------.........I.TT..
AF361083*     ...............I.TT..
AF334619      ------.........I.TT..
AF334620      ------.L.............
AF361082      ...............I.TT..
AF408151      ...............I.TT..
AF408152      ---............I.TT..
AF408153      ...............I.TT..
AF408150*     ...............I.TT..
AY728187      ...............I.TT..
EU419033*     ---............I.TT..
EU419034      ---..................
EU419035      ---............I.TT..
EU419036      ---............I.TT..
EU419037*     ---..................
EU419044      ---....L.............
EU419046      ---............I.TT..
EU419032*     ---............I.TT..
EU688987      ---............I.TT..
FJ562108      ---------------------
GU112267      ...............I.TT..
GU112292      ...............I.TT..
GU112321      ...............I.TT..
GU112324      ...............I.TT..
3DL01CONS     ---............I.TT..

AY728188      .....L...........T...
AY505478      ---------------------
EU419050      ---I.L...........T...
EU419052*     ---I.L...........T...
EU419054      ---I.L...........T...
EU688989      ---I.L...........T...
GU112277      ...I.L...........T...
GU112281      ...I.L...........T...
3DL02CONS     ---I.L...........T...

EU419051      ---I.L...........T...
EU419055      ---I.L...........T...
EU419031      ---I.L...........T...
3DLW03CONS    ---I.L...........T...

EU419040*     .......L.............
EU419042      ---....L.............
3DL04CONS     ---....L.............

EU419045      ---....L.............
EU419061      ---..................
EU419062      ---..L...............
EU419066      ---..L...............
EU419067      ---..L.L.........M...
EU419069      ---....L..F..........
EU688991*     .....L...............
FN424252      .....................
GU112291      .....L...............
GU112310      ---....L..F..........
3DL05CONS     ---..L...............

AF334621      ------.L..F...W......
EU419056      ---....L..F...W......
3DL06*002     ---....L..F...W......

AF334622      ------.L..F..........
AF361086      .......L..F.L........
EU419057      ---....L..F..........
EU419060      ---....L..F..........
EU419063      ---....L.............
EU419064      ---..L.L....L........
EU419065      ---....L..F..........
EU419068      ---....L..F...W......
EU688990*     .......L..F..........
EU688992      ---....L..F...W......
3DL07CONS     ---....L..F..........

EU419071*     -......L.............
AF361084*     .......L.............
AF361085      .......L.............
AY505477      ---------------------
AY728189      -......L.............
EU419070      ---....L.............
GU112268      .......L.............
GU112285      .......L.............
GU112290      .......L.............
GU112330      .......L.............
3DL08CONS     ---....L.............

AY728183      .....L...............
AF334624*     ------..V.........T..
EU419038      ---..L...............
EU419039      ---..................
GU112295      .....L...............
3DL10CONS     ---..L...............

AF334626*     ------..V...L.W......
FN424250      ............L........
FN424259      ............L........
FN424261      ............L........
GU112276      .....................
GU112296      .....................
3DL11CONS     ............L........
```

```
AF361087*      .......L.............
AY728190       .L.....L.............
EU702470       ---------------------
3DS01CONS      .......L.............

AF334649       ------.L.........M...
AY505483       ...T...L.........M...
AY505484       .L.....L.........M...
EU419026*      ---....L.........M...
EU419027       ---....L.........M...
EU702462       ---------------------
EU702461       ---------------------
EU702456*      .....L.L.........M...
GU112261*      .....L.L.........M...
GU112297       .....L.L.........M...
GU112323       .....L.L.........M...
3DS02CONS      ---....L.........M...

AF334650*      .....................
EU702455       .....................
EU702454       ---------------------
3DS03*00101    ------...............

AF344651       ------...............
EU419028       ---..................
EU419029       ---..................
EU702452       ---------------------
3DS04CONS      ---..................

EU419024       ---I.L...............
EU419025*      ---I.L...............
FN424260       ...I.L...............
3DS05CONS      ---I.L...............

AY505485       .L.............I.....
EU688985*      ...I...........I.....
EU702464       ---------------------
FN424257       ...I...........I.....
GU112260       ...I...........I.....
GU112314       ...I...........I.....
3DS06*002      ...I...........I.....

EU702453*      .....L...............
FN424258       .....L...............
3DSW07*001     ---------------------

AY505479       .L.....L..F..........
AY505480       ...T...L..F..........
AY505481       .L.T...L..F..........
AY505482       .L.T...L..F..........
EU702467       ---------------------
FN424254       ...I.L........W......
FN424255       ...I.L........W......
GU112325       ...I.L........W......
GU112328       .......L..F..........
3DSW08*001     .L.....L..F..........

EU419030       ---....L....L........
EU688986       ---..L.L....L........
GU112301       .....L.L....L........
EU702466*      ............L.W......
FN424249       ...I.L...............
3DSW09CONS     ---..L.L....L........
```

D0 DOMAIN

```
                     30        40        50        60        70        80        90       100       110       120
              ...|....|....|....|....|....|....|....|....|....|....|....|....|....|....|....|....|....|....|....|.
Consensus     HTGGQDKTFLSARPSALVPQGGHVTLRCHYRRGLNNFTNFTLYKDDRSHVPIFHSRIFQESFLMGPVTPAHAGTYRCRGSYPHSPTEWSALSDPLAIRVT

AF334616      Y.......S.........................Y..................PG.............................................
AF334617      Y.......S.........................Y..................PG...............D.....................E..T.M..
AF361083*     ........S.........................Y..................PG...............D.....................E....M..
AF334619      ........S.........................Y..................PG...............D.....................E....M..
AF334620      ..........F.....V...........Y..D...................V................................................
AF361082      ........S.........................Y..................PG...............D.....................E....M..
AF408151      ........S.........................Y..................PG...............D.....................E....M..
AF408152      ........S.........................Y..................PG...............D.....................E....M..
AF408153      ........S.........................Y..................PG...............D.....................E....M..
AF408150*     ........S.........................Y..................PG...............D.....................E....M..
AY728187      Y.......S.........................Y..................PG.............................................
EU419033*     Y.......S.........................Y..................PG.............................................
EU419034      ..................................Y..................PG.............................................
EU419035      Y.......S.........................Y..................PG.............................................
EU419036      Y.......S.........................Y..................PG.............................................
EU419037*     ..................................Y..................PG.............................................
EU419044      ........S.................Q.......Y..................LG...............D..........................M..
EU419046      Y.......S.........................Y..................PG...............D.....................E..T.M..
EU419032*     Y.......S.........................Y..................PG.............................................
EU688987      Y.......S.........................Y..................PG.............................................
FJ562108      Y.................................Y..................PG.............................................
GU112267      ........S.........................Y..................PG...............D.....................E....M..
GU112292      ........S.........................Y..................PG...............D.....................E....M..
GU112321      ........S.........................Y..................PG...............D.....................E....M..
GU112324      ........S......................H..Y..................PG...............D.....................E....M..
3DL01CONS     ........S.........................Y..................PG...............D.....................E....M..

AY728188      Y......N....W..................G.---.N..............................................L....TP.N.......
AY505478      -......N....W..................G.---.N..............................................L....TP.N.......
EU419050      Y......N....W..................G.---.N..............................................L....TP.N.......
EU419052*     Y......N....W..................G.---.N..............................................L....TP.N.......
EU419054      Y......N....W..................G.---.N..............................................L....TP.N.......
EU688989      Y......N....W..................G.---.N..............................................L....TP.N.......
GU112277      Y......N....W..................G.---.N..............................................L....TP.N.......
GU112281      Y......N....W..................G.---.N..............................................L....TP.N.......
3DL02CONS     Y......N....W..................G.---.N..............................................L....TP.N.......

EU419051      Y......N....W..PV..............G.---.N...........I.......................................TP.N.......
EU419055      Y.....N.....W..PV..............G.---.N.............V.....................................TP.N.......
EU419031      Y.....N.....W..PV..............G.---.N...........I..................................................
3DLW03CONS    Y.....N.....W..PV..............G.---.N...........I.......................................TP.N.......

EU419040*     ............................Y.....H..................PG...HQ..........D..........Q..........E....M..
EU419042      ............................Y.....H..................PG...HQ..........D..........Q..........E....M..
3DL04CONS     ............................Y.....H..................PG...HQ..........D..........Q..........E....M..

EU419045      Y.......S...Q...............Y....F.................V......HQ........................................
EU419061      .......I........V...........Y..D.................................T..................................
EU419062      .......I........V...........Y..D....................................................................
EU419066      .......I........V...........Y..D....................................................................
EU419067      ..........F.....V...........Y..D.................................................................M..
EU419069      Y..............PV....R.........D...................V.......K.....................................M..
EU688991*     .......I........V...........Y..D.................................T..................................
FN424252      .......I........V...........Y..D....................................................................
GU112291      .......I........V...........Y..D....................................................................
GU112310      Y..............PV....R.........D...................V.......K.....................................M..
3DL05CONS     .......I........V...........Y..D....................................................................

AF334621      ........S.......V.................Y................V................Q............................M..
EU419056      ........S.......V.................Y................V................Q............................M..
3DL06*002     ........S.......V.................Y................V................Q............................M..

AF334622      ..........F.....V...........Y..D....................................................................
AF361086      ..........F.....V...........Y..D...................V................................................
EU419057      ..........F.....V...........Y..D..................................M..............................M..
EU419060      ..........F.....V...........Y..D....................................................................
EU419063      ..........F.....V...........Y..D....................................................................
EU419064      ...D......F.....V...........Y..D....................................................................
EU419065      ..........F.....V...........Y..D...................V................................................
EU419068      ..........F.....V...........Y..D...................V................................................
EU688990*     ..........F.....V...........Y..D..................................M..............................M..
EU688992      ..........F.....V...........Y..D...................V................................................
3DL07CONS     ..........F.....V...........Y..D....................................................................

EU419071*     Y.......S...Q....................F.................V......HQ...................................V....
AF361084*     Y.......S...Q...............Y....F.................V......HQ........................................
AF361085      Y.......S...Q...............Y....F.................V......HQ........................................
AY505477      ----....S.........................Y................V......HQ.....................................M..
AY728189      Y.......S...Q....................F.................V......HQ...................................V....
EU419070      Y.......S...Q....................F.................V......HQ...................................V....
GU112268      Y.......S...Q...............Y....F.................V......HQ........................................
GU112285      ........S...Q...............Y....F.................V......HQ........................................
GU112290      Y.......S...Q...............Y....F.................V......HQ........................................
GU112330      ........S...Q...............Y....F.................V......HQ.....................................M..
3DL08CONS     Y.......S...Q...............Y....F.................V......HQ........................................

AY728183      .......I........V...........Y..D.................................T..................................
AF334624*     Y..........VQ...............Y....F....................N....Q..........D..........................M..
EU419038      .......I........V...........Y..D.................................T..................................
EU419039      .......I........V...........Y..D.................................T..................................
GU112295      .......I........V...........Y..D.................................T..................................
3DL10CONS     .......I........V...........Y..D.................................T..................................

AF334626*     ........S...W..PV.........Q......F...................PG...HQ.....................................M..
FN42450       ................V.........Q......F...................LG........I.................................M..
FN424259      ................V.........Q......F...................LG........I.................................M..
FN424261      ................V.........Q......F...................L.........I.................................M..
GU112276      ................V.........Q......F...................LG........I.................................M..
GU112296      ................V.........Q......F...................LG........I.................................M..
3DL11CONS     ................V.........Q......F...................LG........I.................................M..
```

```
AF361087*     ................V........FQ.Y.H............................Q........................................
AY728190      ................V........FQ.Y.H............................Q........................................
EU702470      ---.............V........FQ.Y.H.....................................................................
3DS01CONS     ................V........FQ.Y.H............................Q........................................

AF334649      ..........F.....V...........Y..D..................................M..............................M..
AY505483      ..........F.....V...........Y..D..................................M.............................VM..
AY505484      ..........F.....V...........Y..D.................................................................M..
EU419026*     ..........F.....V...........Y..D.................................................................M..
EU419027      ..........F.....V...........Y..D.................................................................M..
EU702462      ---.......F.....V...........Y..D.................................................................M..
EU702461      ---.......F.....V...........Y..D.................................................................M..
EU702456*     ..........F.....V...........Y..D..................................M..............................M..
GU112261*     ..........F.....V...........Y..D.................................................................M..
GU112297      ..........F.....V...........Y..D.................................................................M..
GU112323      ..........F.W...V...........Y..D.................................................................M..
3DS02CONS     ..........F.....V...........Y..D.................................................................M..

AF334650*     .......I...VQ..........M..........Y...................N...............D..........................M..
EU702455      .......I...VQ..........M..........Y...................N...............D..........................M..
EU702454      ---....I...VQ..........M..........Y...................N...............D..........................M..
3DS03*00101   .......I...VQ..........M..........Y...................N...............D..........................M..

AF344651      ......N...................Q......F.................V.......Q.....................................M..
EU419028      ......N...................Q......F.................V.......Q.....................................M..
EU419029      ......N...................Q......F.................V.......Q.....................................M..
EU702452      ---...N...................Q......F.................V.......Q.....................................M..
3DS04CONS     ......N...................Q......F.................V.......Q.....................................M..

EU419024      ......NA....W..PV..............G.---.N...........L........................................P.N.......
EU419025*     ......NA....W..PV..............G.---.N....................................................P.N.......
FN424260      ......NA....W..PV..............G.---.N....................................................P.N.......
3DS05CONS     ......NA....W..PV..............G.---.N...........L........................................P.N.......

AY505485      ...A...N..F.W..PV.....L.....Y.H..................................................................M..
EU688985*     ...A...N..F.W..PV.....L.....Y.H..................................................................M..
EU702464      ---A...N..F.W..PV.....L.....Y.H..................................................................M..
FN424257      .......N..F.W..PV.....L.....Y.H..................................................................M..
GU112260      ...A...N..F.W..PV.....L.....Y.H..................................................................M..
GU112314      ...A...N..F.W..PV.....L.....Y.H..................................................................M..
3DS06*002     ...A...N..F.W..PV.....L.....Y.H..................................................................M..

EU702453*     ............................Y..D...................V..N.............................................
FN424258      ............................Y..D...................V..N.............................................
3DSW07*001    --..........................Y..D...................V..N.............................................

AY505479      ........S..........................................V.........................................H......
AY505480      ........S..........................................V................................................
AY505481      ........S..........................................V................................................
AY505482      ........S..........................................V................................................
EU702467      ---.....S..........................................V................................................
FN424254      ........S.......V..................................V................Q..T....................E....M..
FN424255      ..................................Y................V................Q..T....................E....M..
GU112325      ........S.......V..................................V................Q..T....................E....M..
GU112328      ........S..........................................V................................................
3DSW08*001    ........S..........................................V................................................

EU419030      ...D......F.....V...........Y..D....................................................................
EU688986      ...D......F.....V...........Y..D....................................................................
GU112301      ...D......F.....V...........Y..D....................................................................
EU702466*     .......I........V...........Y..D....................................................................
FN424249      ........S..........................................V................Q..T....................E....M..
3DSW09CONS    ...D......F.....V...........Y..D....................................................................
```

D1 DOMAIN

```
                      130       140       150       160       170       180       190       200       210       220
              ...|....|....|....|....|....|....|....|....|....|....|....|....|....|....|....|....|....|....|....|.
Consensus     GVHRKPSLLALPGPLVKSGETVTLQCSSDTVFEHFFLHSEVTFEEPLHLVGELHGGGSQANYSINSTTSDLAGTYRCYGSVTHSPYVLSAPSDPLDIVIT

AF334616      ...K..................................R.............R......V............E...........................
AF334617      ......................................R.............R......V....................A...................
AF361083*     ...K..................................R.............R......V............E.......A...................
AF334619      ...K..................................R.............R......V............E.......A...................
AF334620      ......................................R.............R......V....................T...................
AF361082      ...K..................................R.............R......V............E.......A...................
AF408151      ...K..................................R.............R......V............E.......X...................
AF408152      ...K..................................R............AX......V............E.......A...................
AF408153      ...K...........................X......R.............R......V............E.......A...................
AF408150*     ...K..................................R.............R......V............E.......A................M..
AY728187      ...K..................................R.............R......V............E.......A...................
EU419033*     ...K..................................R.............R......V............E...........................
EU419034      ...K..................................R.............R......V............E.......A...................
EU419035      ...K..................................R.............R......V............E.......A...................
EU419036      ...K..................................R.............R......V............E.......A...................
EU419037*     ...K..................................R.............R......V............E.......A...................
EU419044      ...K..................I....................Q........R...S..V....................A...................
EU419046      ......................................R.............R......V....................A...................
EU419032*     ...K..................................R.............R......V............E.......A...................
EU688987      ...K..................................R.............R......V............E.......A...................
FJ562108      ...K..................................R.............R......V............E.......A...................
GU112267      ...K..................................R.............R......V............E.......A...................
GU112292      ...K..................................R.............R......V............E.......A...................
GU112321      ...K..................................R.............R......V............E.......A................M..
GU112324      ...K..................................R.............R......V............E.......A................M..
3DL01CONS     ...K..................................R.............R......V............E.......A...................

AY728188      ...K..................................R...L.......................MM......F.....F...................
AY505478      ...K..................................R...L.......................MM......F.....F...................
EU419050      ...K..................................R...L........K..............MM......F.....F...................
EU419052*     ...K..................................R...L.......................MM......F.....F...................
EU419054      ...K..................................R...L........K..............MM......F.....F...................
EU688989      ...K..................................R...L........K..............MM......F.....F...................
GU112277      ...K..................................R...L........K..............MM......F.....F...................
GU112281      ...K........................................K.....................MM......F.....F...................
3DL02CONS     ...K..................................R...L........K..............MM......F.....F...................

EU419051      ...K..................................R...L.......E.........K....................F...................
EU419055      ...K..................I...............R...L.............S.........MMYT....F.....F...................
EU419031      ...K..................................R...L.................K....................F...................
3DLW03CONS    ...K..................................R...L.................K....................F...................

EU419040*     .............................I..G............L......................................................
EU419042      .............................I..D............L......................................................
3DL04CONS     .............................I..D............L......................................................

EU419045      .....................................Q.....KKSV................MGP...A..............................
EU419061      .....................................Q.....KKSV................MGP...A..............................
EU419062      .....................................Q.....KKSV................MGP...A..............................
EU419066      ...........................................KKSV................MGP...A..............................
EU419067      ................................G...........K.......R.............KM................H...............
EU419069      .....................................QN....KKS.................MGP...A........................S......
EU688991*     .....................................Q.....KKSV................MGP...A..............................
FN424252      .....................................Q.....KKSV................MGP...A..............................
GU112291      .....................................Q.....KKSV................MGP...A..............................
GU112310      .....................................QN....KKS.................MGP...A..............D.........S......
3DL05CONS     .....................................Q.....KKSV................MGP...A..............................

AF334621      ........................................................................E.L....................N....
EU419056      ........................................................................E.L....................N....
3DL06*002     ........................................................................E.L....................N....

AF334622      .............................M...........N..K.......................................D...............
AF361086      .........................................N..K.......................................D...............
EU419057      ......................I..................N..K.......................................D...............
EU419060      .........................................N..K.......................................D...............
EU419063      .............................M...........N..K.......................................D...............
EU419064      .........................................N..K.......................................................
EU419065      ...K.........................M...........N..K.......................................D...............
EU419068      .............................M..G........N..K.......................................................
EU688990*     .........................................N..K.......................................D...............
EU688992      .............................M..G........N..K.......................................................
3DL07CONS     .........................................N..K.......................................D...............

EU419071*     ..........F...........I......I..G............L................................................T......
AF361084*     ..........F.....................G............L................................................T......
AF361085      ..........F.....................G............L.........................P......................T......
AY505477      .............................I..G............L................................................T......
AY728189      ..........F...........I......I..G.........L..L................................................T......
EU419070      ..........F.....................G............L................................................T......
GU112268      ..........F...........I......I..G............L................................................T......
GU112285      ..........F.....................G............L................................................T......
GU112290      ..........F.....................G............L................................................T......
GU112330      ..........F.....................G............L................................................T......
3DL08CONS     ..........F.....................G............L................................................T......

AY728183      ......................I.....................K..........................EE.F....................N....
AF334624*     .............................I..............K..........................EE.F....................N....
EU419038      ....................M.......................K..........................EE.F....................N....
EU419039      ......................I.....................K..........................EE.F....................N....
GU112295      ............................................K..........................EE.F....................N....
3DL10CONS     ............................................K..........................EE.F....................N....

AF334626*     ................................G............L..........................E.F....................N....
FN42450       ................................G............L......................Y...E.F....................N....
FN424259      ....................M...........G............L..........................E.F....................N....
FN424261      ....................M...........G............L..........................E.F....................N....
GU112276      ...K............................G............L..........................E.F....................N....
GU112296      ................................G............L..........................E.F....................N....
3DL11CONS     ................................G............L..........................E.F....................N....
```

```
AF361087*     ................................G...........K.......................................................
AY728190      ................................G...........K.......................................................
EU702470      ............................................K....................................I..................
3DS01CONS     ................................G...........K.......................................................

AF334649      ................................G...........K.......R.............KM................H...............
AY505483      ............................................K.......R.............KM................H...............
AY505484      ................................G...........K.......R.............KM................H...............
EU419026*     ................................G...........K.......R.............KM................H...............
EU419027      ................................G...........K.......R.............KM................H...............
EU702462      ................................G...........K.......R.............KM................H...............
EU702461      ................................G...........K.......R.............KM................H...............
EU702456*     ............................................K.......R.............KM................H...............
GU112261*     ................................G...........K.......R.............KM................H...............
GU112297      ..........F.....................G...........K.......R.............KM................H...............
GU112323      ................................G...........K.......R.............KM................H...............
3DS02CONS     ................................G...........K.......R.............KM................H...............

AF334650*     .........................................N..K.....................K.....E...........................
EU702455      ................................G...........K.....................K.....E............D..............
EU702454      ................................G...........K.....................K.....E...........................
3DS03*00101   .........................................N..K.....................K.....E...........................

AF344651      ......................I......I..G............L...............................................T......
EU419028      ......................I......I..G............L...............................................T......
EU419029      ......................I......I..G............L...............................................T......
EU702452      ................................G............L...............................................T......
3DS04CONS     ......................I......I..G............L...............................................T......

EU419024      ...K..................................R...L.................K.........F...F.....F...................
EU419025*     ...K..................................R...L.................K.........F...F.....F...................
FN424260      ...K......F...........................R...L.................K.........F...F.....F................M..
3DS05CONS     ...K..................................R...L.................K.........F...F.....F...................

AY505485      .................L.......................N..K.....................K..E........................R.....
EU688985*     .................L.......................N..K.....................K..E........................R.....
EU702464      .................L.......................N..K.....................K..E........................R.....
FN424257      .................L..............D........N..K.....................K...........................R.....
GU112260      .................L.......................N..K.....................K..E........................R.....
GU112314      .................L.......................N..K.....................K..E........................R.....
3DS06*002     .................L.......................N..K.....................K..E........................R.....

EU702453*     ...................D............G...........K.....................K.....E...........................
FN424258      ................................G...........K.....................K.....E...........................
3DSW07*001    ...................D............G...........K.....................K.....E...........................

AY505479      .............................I..G........N...L...............................................T......
AY505480      .............................I..G........N...L................F..............................T......
AY505481      .............................I..G........N...L...............................................T......
AY505482      .............L...............I..G........N...L.....................................P.........T......
EU702467      ..........F.....................G............L....................M..................D.......T......
FN424254      .............................M...............L....................M..................D.......T......
FN424255      ................................G............L....................M..................D.......T......
GU112325      ................................G............L....................M..................D.......T......
GU112328      .............................I..G........N...L...............................................T......
3DSW08*001    .............................I..G........N...L...............................................T......

EU419030      .............................I...........N..K.......................................................
EU688986      ......................I..................N..K.......................................................
GU112301      .........................................N..K.......................................................
EU702466*     .............................M...........N..K................................................T......
FN424249      .............................M...........N..K.....................K..........................T......
3DSW09CONS    .........................................N..K.......................................................
```

D2 DOMAIN

```
                  230       240       250       260       270       280       290       300       310
              ...|....|....|....|....|....|....|....|....|....|....|....|....|....|....|....|....|....|....
Consensus     GLYEKPSLSAQPGPTVQAGENVTLSCSSQ-SFDMYHLSREGEARELSLSAVPSVNGTFQADFPLGPATHGGTYRCFGSFRTAPYKWSDPSDPLPVSVT
AF334616      .............................T.............N..R.P..S........N.L...............Y.DS..E........S....
AF334617      .............................T..H..........N..R.P..S........N.L...............Y.DS..E........S....
AF361083*     .............................T.............N..R.P..S........N.L...............YHDS..E........S....
AF334619      .............................T.............N..R.P..S........N.L...............YHDS..E........S....
AF334620      .............................T..H..........N..R.P..S........N.L...............Y.DS..E........S....
AF361082      .............................T.............N..R.P..S........N.L...............YHDS..E........S....
AF408151      .............................T.............N..R.P..S........N.L...............YHDS..E........S....
AF408152      .............................T.............N..R.P..S........N.L...............YHDS..E........S....
AF408153      .............................T.............N..R.P..S........N.L...............YHDS..E........S....
AF408150*     .............................T..H..........N..R.P..S........N.L...............Y.DS..E........S....
AY728187      .............................T.............N..R.P..S........N.L...............Y.DS..E........S....
EU419033*     .............................T.............N..R.P..S........N.L...............Y.DS..E........S....
EU419034      .............................T.............N..R.P..S........N.L...............Y.DS..E........S....
EU419035      .............................T.............N..R.P..S........N.L...............Y.DS..E........S....
EU419036      .............................T.............N..R.P..S........N.L...............Y.DS..E........S....
EU419037*     .............................T.............N..R.P..S........N.L...............Y.DS..E........S....
EU419044      .............................T.............N..R.P..S........N.L...............Y.DS..E........S....
EU419046      .............................T..H..........N..R.P..S........N.L...............Y.DS..E........S....
EU419032*     .............................T.............N..R.P..S........N.L...............Y.DS..E........S....
EU688987      .............................T.............N..R.P..S........N.L...............Y.DS..E........S....
FJ562108      .............................T.............N..R.P..S........N.L...............Y.DS..E........S....
GU112267      .............................T.............N..R.P..S........N.L...............YHDS..E........S....
GU112292      .............................T.............N..R.P..S.....L..N.L...............YHDS..E........S....
GU112321      .............................T..H..........N..R.P..S........N.L...............Y.DS..E........S....
GU112324      .............................T..H..........N..R.P..S........N.L...............Y.DS..E........S....
3DL01CONS     .............................T.............N..R.P..S........N.L...............Y.DS..E........S....

AY728188      .K.............I............RC......................................................Q.............
AY505478      .K.........L.................N....................................................................
EU419050      .K...........................N....................................................................
EU419052*     .K.............I............RC......................................................Q.............
EU419054      .K.............I............RC......................................................Q.............
EU688989      .K.............I............RC......................................................Q.............
GU112277      .K.............I............RC......................................................Q.............
GU112281      .K.............I............RC......................................................Q.............
3DL02CONS     .K.............I............RC......................................................Q.............

EU419051      ............................RC.............................G...........................H..........
EU419055      ............................RC.....................R.......G...........................H..........
EU419031      ............................RC.....................R.......G....................N......H..........
3DLW03CONS    ............................RC.....................R.......G...........................H..........

EU419040*     .K...........................N.............................S........................Q........H....
EU419042      .K...........................N.............................S........................Q........H....
3DL04CONS     .K...........................N.............................S........................Q........H....

EU419045      .I.K........................RR............TH..R.P.................V.........A.......E..V.....H..I.
EU419061      .I.K........................RR............TP..R.P.................V.........A.......E..V.....H..I.
EU419062      .I.K........................RR............TH..R.P.................V.........A.......E..V.....H..I.
EU419066      .I.K........................RR............TH..R.P.................V.........A.......E..V.....H..I.
EU419067      .I.K........................RR............TH..R.P.................V.........A.......E..V.....H....
EU419069      .I.K........................WR............TH..R.P.................V.................E..V.....H....
EU688991*     .I.K........................RR............TH..R.P.................V.........A.......E..V.....H..I.
FN424252      .I.K........................RR............TH..R.P.................V.........A.......E..V.....H..I.
GU112291      .I.K........................RR............TH..R.P.................V.........A.......E..V.....H..I.
GU112310      .I.K........................WR............TH..R...................V.................E..V.....H....
3DL05CONS     .I.K........................RR............TH..R.P.................V.........A.......E..V.....H..I.

AF334621      .K...........................N.......Y......................N..................C.......H..........
EU419056      .............................N.......Y......................N..................C.......H..........
3DL06*002     .............................N.......Y......................N..................C.......H..........

AF334622      .K...........................N....................................................................
AF361086      .K.......S............I.....RC......................................................Q.............
EU419057      .K...........................N....................................................................
EU419060      .K...........................N....................................................................
EU419063      .K....................I.....RC............................K.........................Q.............
EU419064      .K...........................N....................................................................
EU419065      .K...........................N....................................................................
EU419068      .K....................I.....RC..................................................S...Q.............
EU688990*     .K...........................N....................................................................
EU688992      .K....................I.....RC..................................................S...Q.............
3DL07CONS     .K...........................N....................................................................

EU419071*     ................H.....I.....RC............T............................................H..........
AF361084*     ................H.....I.....RR............T............................................H..........
AF361085      ................H.....I.....RR............T............................................H..........
AY505477      ................H.....I.....RR.........Q..T............................................H..........
AY728189      ................H.....I.....RC............T............................................H..........
EU419070      ................H.....I.....RC............T............................................H..........
GU112268      ................H.....I.....RR............T............................................H..........
GU112285      ................H.....I.....RC............T............................................H..........
GU112290      ................H.....I.....RR............T............................................H..........
GU112330      ................H.....I.....RR.........Q..T............................................H..........
3DL08CONS     ................H.....I.....RR............T............................................H..........

AY728183      .K...........................N............S....V...R.......G...........I........IT.....NS....L....
AF334624*     .K...........................N............S....V...R.......G...........I........IT.....NS....L....
EU419038      .K...........................N............S....V...R.......G...........I........IT.....NS....L....
EU419039      .K...........................N............S....V...R.......G...........I........IT.....NS....L....
GU112295      .K...........................N............S....V...R.......G...........I........IT.....NS....L....
3DL10CONS     .K...........................N............S....V...R.......G...........I........IT.....NS....L....

AF334626*     .K....................I.....RC....................................................................
FN42450       .K...........L........I.....RC.......Y.....H.....................................................K
FN424259      .K....................I.....RC....................................................................
FN424261      .K....................I.....RC....................................................................
GU112276      .K.............I............RC......................................................Q.............
GU112296      .K....................I.....RC.......................I............................................
3DL11CONS     .K....................I.....RC....................................................................
```

```
AF361087*    ....................D........I..................................................S...Q........HI...
AY728190     ....................D........I..................................................S...Q........HI...
EU702470     ....................D........I..................................................S...Q........HI...
3DS01CONS    ....................D........I..................................................S...Q........HI...

AF334649     ............................RR.....................R.......G...................H.T.....H..........
AY505483     ............................RR.....................R.......G...................H.T.....H..........
AY505484     ............................RR.....................R.......G...................H.T.....H..........
EU419026*    ............................RR.....................R.......G...................H.T.....H..........
EU419027     ............................RR.....................R.......G...................H.I.....H..........
EU702462     .............................N..................A..R.......G...................H.T.....H..........
EU702461     ............................RR.....................R.......G...................H.T.....H..........
EU702456*    ............................RR.....................R.......G...................H.T.....H..........
GU112261*    ............................RR.....................R.......G...................H.T.....H..........
GU112297     ............................RR.....................R.......G...................H.T.....H..........
GU112323     ............................RR.....................R.......G...................H.T.....H..........
3DS02CONS    ............................RR.....................R.......G...................H.T.....H..........

AF334650*    .K...........................N..............G......Q..............................................
EU702455     .K...........................N..............G......Q..............................................
EU702454     .K...........................N..............G......Q..............................................
3DS03*00101  .K...........................N..............G......Q..............................................

AF344651     .K..........................RC...............I...................................T...............K
EU419028     .K..........................RC...............I...................................T...............K
EU419029     .K..........................RC...............I...................................T...............K
EU702452     .K..........................RC...............I...................................T...............K
3DS04CONS    .K..........................RC...............I...................................T...............K

EU419024     ............................RR.............................-.....................TS....H..........
EU419025*    ............................RR.............................-.....................TS....H..........
FN424260     ............................RR.............................-.....................TS....H..........
3DS05CONS    ............................RR.............................-.....................TS....H..........

AY505485     .............................N..................A..R.......G...................H.T.....H..........
EU688985*    .............................N..................A..R.......G...................H.T.....H..........
EU702464     ................H............N..................A..R.......G...................H.T.....H..........
FN424257     ............................RC..................A..R.......G...................H.T.....H..........
GU112260     .............................N..................A..R.......G...................H.T.....H..........
GU112314     .............................N..................A..RR......G...................H.T.....H..........
3DS06*002    .............................N..................A..R.......G...................H.T.....H..........

EU702453*    .K...........................N..............G......Q...R............................Q.............
FN424258     .K...........................N..............G......Q..............................................
3DSW07*001   .K...........................N..............G......Q...R............................Q.............

AY505479     .K...........................N..............G......Q........N.....................................
AY505480     .K...........................N..............G......Q........N.....................................
AY505481     .K...........................N..............G......Q........N.....................................
AY505482     .K...........................N.....................R.....S.G...................H.T.....H..........
EU702467     .............................N..............G......Q...R............................Q........S....
FN424254     .............................N..............G......Q...R............................Q........S....
FN424255     .............................N..............G......Q...R............................Q........S....
GU112325     .............................N..............G......Q...R............................Q........S....
GU112328     .K...........................N..............G......Q........N.....................................
3DSW08*001   .K...........................N..............G......Q........N.....................................

EU419030     ............................RC.....................R.......G...........................H..........
EU688986     ............................RC.....................R.......G...........................H..........
GU112301     ............................RC.....................R.......G...........................H..........
EU702466*    ............................RC............T........R..............................................
FN424249     ............................RC.V..........T........R..............................................
3DSW09CONS   ............................RC.....................R.......G...........................H..........
```

```
STEM, TRANSMEMBRANE, CYTOPLASMIC TAIL
              320       330       340       350       360       370       380       390       400        410       420       430       440
              |....|....|....|....|....|....|....|....|....|....|....|....|....|....|....|....|....|....|....|....|....|....|....|....|....
Consensus     GNPSRSWPSPTEPSSKTSIPRHL--HVLIGTSVVMILFTI-FFFLLHRWCSNKKNAAVMDQEPAGDRTVNREDSDEQDPQEVTYAQLDHRVLTQGKITRPSQRPKTPPTDTSVYTELPNAEPRSKVVFYP

AF334616      ..................N....--........A......-................................P........................C.....RR........................
AF334617      ..................N....--........A......-................................P...............C........C.....RR........................
AF361083*     ..................N....--........A......-......C.........A............P...E.......................................................
AF334619      ..................N....--...............-.................I...............E.......................H......................S.W...SC.
AF334620      ..................N....--........A......-................................P...............C......T.................................
AF361082      ..................N....--........A......-................................P........................C.....RR........................
AF408151      ..................N....--........A......-......C.........A............P...E.......................................................
AF408152      ..................N....--........A......-......C.........A............P...E.......................................................
AF408153      ..................N....--........A......-......C.........A............P...E.......................................................
AF408150*     ..................N....--........A......-................................P........................C.....RR........................
AY728187      ..................N....--........A......-................A............P...E.......................................................
EU419033*     ..................N....--........A......-................................P...............C........................................
EU419034      ..................N....--........A......-................................P...............C........................................
EU419035      ..................N....--........A......-......C.........................P........................C.....RR........................
EU419036      .......................--........A......-................................P........................C.....RR........................
EU419037*     ..................N....--........A......-................................P........................C.....RR........................
EU419044      ....H.............N....--...............-.........................T.........P.....................H...............................
EU419046      ..................N....--........A......-................................P........................C.....RR........................
EU419032*     ..................N....--........A......-................................P........................C.....RR........................
EU688987      .......................--........A......-................................P...............C........................................
FJ562108      ..................N....--........A......-.......
GU112267      ..................N....--........A......-......C.........................P........................C.....RR........................
GU112292      ..................N....--........A......-......C.........A............P...E.......................................................
GU112321      ..................N....--........A......-......C.........................P........................C.....RR........................
GU112324      ..................N....--........A......-......C.........................P........................C.....RR........................
3DL01CONS     ..................N....--........A......-................................P........................C.....RR........................

AY728188      .....T.................--.........T.....-......L.........A............P...........................................................
AY505478      .....T.................--...............-................A............P........................................................X--
EU419050      .......................--...............-................A............P...........................................................
EU419052*     .....T.................--.........T.....-......L.........A............P...........................................................
EU419054      .......................--........A......-................................P........................C.....RR........................
EU688989      .......................--.........T.....-......L.........................P..............................RR........................
GU112277      .......................--.........T.....-......L......................P................................................P..........
GU112281      .....T.................--.........T.....-......L......................P................................................P..........
3DL02CONS     .......................--.........T.....-......L.........A............P...........................................................

EU419051      .............N...G.....--........I......-................................P...............C........H...K...........R...............
EU419055      .............N...G.....--...............-................................P........................H...K.........M.R...............
EU419031      .............N...G.....--...............-................................P........................H...K...........I...............
3DLW03CONS    .............N...G.....--...............-................................P........................H...K...........R...............

EU419040*     ..................N....--........A......-...................................P.....................................................
EU419042      ..................N....--........A......-...................................P.....................................................
3DL04CONS     ..................N....--........A......-...................................P.....................................................

EU419045      ....S..................--...............-................................P............................K...........................
EU419061      .......................--...............-................A............P...........................................................
EU419062      .......................--...............-................A............P...........................................................
EU419066      .......................--...............-.................K...............E....L..................H...K...........................
EU419067      ....S........N.........--...............-................A................E.........T.............................................
EU419069      ....S..................--........A......-................................................C.F..E..........R........IK.....S.W...SC.
EU688991*     .......................--...............-.................K...............E.......................H...K...........................
FN424252      ....S..................--...............-................................P........................H...K...........................
GU112291      .......................--........A......-.................K...............E....L..................H...K...........................
GU112310      ....S..................--........A......-................................................C.F..E..........R........IK.....S.W...SC.
3DL05CONS     .......................--...............-.........................................................................................

AF334621      ..I..............GN....--........A......-................A................E.........T.............................................
EU419056      ..I...............N....--........A......-................A................E.......................................................
3DL06*002     ..I...............N....--........A......-................A................E.......................................................

AF334622      .......................--...............-................A............P...........................................................
AF361086      .................GN....--...............-.................I...............E.......................H......................S.W...SC.
EU419057      .......................--...............-................A............P...........................................................
EU419060      .......................--...............-................A............P...........................................................
EU419063      ..................N....--...............-..................N......................................H...............................
EU419064      ..................N....--........A......-................................P........................................................
EU419065      .................G.....--...............-................A............P...E.......................................................
EU419068      ..................N....--...............-..................................................S......................................
EU688990*     .......................--...............-................A............P...........................................................
EU688992      ..................N....--...............-.................K...............E....L..................H...K...........................
3DL07CONS     ..................N....--...............-................A........................................................................

EU419071*     .......................--...............-.................K...............E....E.........C......T......R.R........I......S.....SC.
AF361084*     .......................--...............-.................K...............E....E................T......R........M.M......S.....SC.
AF361085      .......................--...............-.................K...............E....E................T......R........M.M......S.....SC.
AY505477      .......................--...............-.................K...............E....E.........C......T......R........M.M......S.-------
AY728189      .......................--...............-.................K...............E....E.........C......T......R.R........I......S.....SC.
EU419070      .......................--...............-.................K...............E....E.........C......T......R.R........I......S.....SC.
GU112268      .......................--...............-.................K...............E....E................T......R........M.M......S.....SC.
GU112285      .......................--...............-.................K...............E....E.........C......T......R.R........I......S.....SC.
GU112290      .......................--...............-.................K...............E....E................T......R..........I......S.....SC.
GU112330      .......................--...............-.................K...............E....E.........C......T......R........M.M......S.....SC.
3DL08CONS     .......................--...............-.................K...............E....E.........C......T......R.R........I......S.....SC.

AY728183      .D..S............G.....--...............-.................K...............E..............C......T......R..........M......S.....SC.
AF334624*     .D..S............G.....--...............-.................K...............E....E.........C......S......R..........M......S.....SC.
EU419038      .D..S............G.....--...............-.................K...............E..............C......T......R..........M......S.....SC.
EU419039      .D..S............G.....--...............-.................K...............E..............C......T......R..........M......S.....SC.
GU112295      .D..S............G.....--...............-.................K...............E..............C......T......R..........M......S.....SC.
3DL10CONS     .D..S............G.....--...............-.................K...............E..............C......T......R..........M......S.....SC.

AF334626*     ..................Y....--........A......-................................P...............C........................................
FN42450       ....H..................--...............-...................................P.....................................................
FN424259      .................GN....--...............-................................P...............C........................................
FN424261      .................GN....--.......A.......-................................P...............C........................................
GU112276      .......................--.........T.....-......L......................P................................................P..........
GU112296      .................GN....--........A......-................................P...............C........C.....RR........................
3DL11CONS     .................GN....--...............-................................P...............C........................................
```

```
AF361087*     ..............C....T...--PIV.RY..AT.I...LL....R....D..RL
AY728190      ..............C....T...--PIV.RY..AT.I...LL....R....D..RL
EU702470      ....S.........C....T...--PIV.RY..AT.I...LL....R....D...........V.TEQ
3DS01CONS     ..............C....T...--PIV.RY..AT.I...LL....R....D..RL

AF334649      ....G........N...G.T...--PIV.RY..AT.I...LL....R....D..RL
AY505483      ....G........N...G.T...--PIV.RY..AT.I...LL....R....D..RL
AY505484      ....G........N...G.T...--PIV.RY..AT.I...LL....RC...D..RL
EU419026*     ....G........N...G.T...--PIV.RY..AT.I...LL....R....D..R
EU419027      ....G........N...G.T...--PIV.RY..AT.I...LL.........D..RL
EU702462      ....S........N...G.T...--PIV.RY..AT.I...LL.........D..RL
EU702461      ....G........N...G.T...--PIV.RY..AT.I...LL....RH...D..RL
EU702456*     ....G........N...G.T...--PIV.RY..AT.I...LL....R....D..RL
GU112261*     ....G........N...G.T...--PIV.RY..AT.I...LL....RC...D..R
GU112297      ....G........N...G.T...--PIV.RY..AT.I...LL....RC...D..RL
GU112323      ....G........N...G.T...--PIV.RY..AT.I...LL....R....D..RL
3DS02CONS     ....G........N...G.T...--PIV.RY..AT.I...LL....R....D..RL

AF334650*     .....T........C..G.T...--PIV.RY..AT.I...LL....R....D..RL
EU702455      .....T........C..G.T...--PIV.RY..AT.I...LL....R....D..RL
EU702454      .....T........C..G.T...--PIV.RY..AT.I...LL....R....D..RL
3DS03*00101   .....T........C..G.T...--PIV.RY..AT.I...LL....R....D..RL

AF344651      ....S.........C....T...--PIV.RY..AT.I...LL....RH...D..RL
EU419028      ....S.........C....T...--PIV.RY..AT.I...LL....R....D..RI
EU419029      ....S.........C....T...--PIV.RY..AT.I...LL....R....D..RL
EU702452      ....S.........C....T...--PIV.RY..AT.FL..LL....R....D..RL
3DS04CONS     ....S.........C....T...--PIV.RY..AT.I...LL....R....D..RL

EU419024      .................G.T.Y.QVPIV.RY..AT.I...LL....R....D......X
EU419025*     .................G.T.Y.QVPIV.RY..AT.I...LL....R....D......X
FN424260      .................G.T.Y.QVPIV.RY..AT.I...LL....R....D...........V.TEQ
3DS05CONS     .................G.T.Y.QVPIV.RY..AT.I...LL....R....D...........V.TEQ

AY505485      ....S........N...G.T...--PIV.RY..AT.I...LL.........D..RL
EU688985*     ....S........N...G.T...--PIV.RY..AT.I...LL....R....D..RL
EU702464      ....S........N...G.T...--PIV.RY..AT.I...LL.........D..RL
FN424257      ....S........N...G.T...--PTV.RY..AT.I...LL....R....D..RL
GU112260      ....S........N...G.T...--PIV.RY..AT.I...LL.........D..RL
GU112314      ....S........N...G.T...--PIV.RY..AT.I...LL....R....D..RL
3DS06*002     ....S........N...G.T...--PIV.RY..AT.I...LL....R....D..RL

EU702453*     ....S.........C..G.T...--PIV.RY..AT.I...LL....R....D..R
FN424258      .....T........C..G.T...--PIV.RY..AT.I...LL.........D..RL
3DSW07*001    ....S.........C..G.T...--PIV.RY..AT.I...LL....R....D..RL

AY505479      .....T........C..G.T...--PIV.RY..AT.I...LL....R....D..RL
AY505480      .....T........C..G.T...--PIV.RY..AT.I...LL....R....D..RL
AY505481      .....T........C..G.T...--PIV.RY..AT.I...LL....R....D..RL
AY505482      ....G........N...G.T...--PIV.RY..AT.I...LL....R....D..RL
EU702467      ....S.........C..G.T...--PIV.RY..AT.I...LL.........D..RL
FN424254      ....S.........C..G.T...--PIV.RY..AT.I...LL....R....D..RL
FN424255      ....S.........C..G.T...--PIV.RY..AT.IS..LL....R....D..RL
GU112325      ....S.........C..G.T...--PIV.RY..AT.I...LL....R....D..RL
GU112328      .....T........C..G.T...--PIV.RY..AT.I...LL....R....D..RL
3DSW08*001    .....T........C..G.T...--PIV.RY..AT.I...LL....R....D..RL

EU419030      ....S.........C....T...--PIV.RY..AT.I...LL....R....D..RL
EU688986      ....S.........C....T...--PIV.RY..AT.I...LL....R....D..RL
GU112301      ....S.........C....T...--PIV.RY..AT.I...LL....R....D..RL
EU702466*     ....S.........C..G.T...--PIV.RY..AT.I...LL.........D..RL
FN424249      ....S.........C..G.T...--PIV.RY..AT.I...LL.........D..RL
3DSW09CONS    ....S.........C....T...--PIV.RY..AT.I...LL....R....D..RL
```
